# Rapid and Efficient Solid‐State Mechanosynthesis of Bipyridine Metal Complexes

**DOI:** 10.1002/chem.202501214

**Published:** 2025-06-23

**Authors:** Talha Munir, Eleonora Aneggi, Walter Baratta, Leonardo Genesin, Daniele Zuccaccia, Fabio Trigatti

**Affiliations:** ^1^ Dipartimento di Scienze Agroalimentari, Ambientali e Animali, Sezione di Chimica Università di Udine Via Cotonificio 108 Udine I‐33100 Italy; ^2^ Dipartimento di Scienze della Vita Università di Trieste Via Weiss 2 Trieste 34128 Italy

**Keywords:** bipyridine, green synthesis, mechanochemistry, metal complexes

## Abstract

2,2′‐bipyridine is considered to be the most widely used chelating ligand and it has been extensively studied especially in the areas of coordination chemistry, medicine, materials, and catalysis. A mechanochemical reaction is a process that involves the application of mechanical energy and it provides a high yield in a shorter reaction time and eliminates the need for bulk solvents, typically request in solvothermal reaction.

Notwithstanding the importance of the synthesis of bipyridine‐metal compounds for several decades and recent advancements in the mechanochemical synthesis the development of a mechanochemical protocol for the synthesis of metal complexes bearing bipyridine remains unexplored.

In this work, we extensively studied the mechanochemistry reaction of more than 10 metallic precursors of Ru, Ir, Pt, Pd, Fe, and Co with 2,2′‐bipyridine in different stoichiometric ratio without utilization of bulk solvents, under ambient conditions and without air‐sensitive procedures. A comparison of the yield, reaction time, temperature, frequency, green metrics (E‐factor and the Effective Mass Yield (EMY)) of conventional solvothermal methodologies and mechanochemical synthesis clearly indicates that the mechanochemical approach is more sustainable and efficient. Mechanochemistry approach can be complementary to common solvothermal approach, and in the future, it will be required greater attention in the field of organometallic chemistry.

## Graphical abstract

The mechanochemistry reaction of more than 10 metallic precursors of Ru, Ir, Pt, Pd, Fe, and Co with 2,2′‐bipyridine afford the target complexes in high yield, in a short reaction time with very low E‐factor and excellent Effective Mass Yield (EMY) values respect to of conventional solvothermal synthesis.

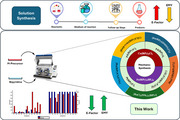

## 1 Introduction

In light of the environmental challenges posed by human activity, researchers are now adapting their methodologies to embrace sustainable and green approaches. For synthetic chemists, the conventional approach involves working with solvents. However, this method is costly, time‐consuming, and most importantly, has significant environmental impacts. As the saying goes, “Everything new is well‐ forgotten old”, mechanochemistry is a vital yet often overlooked approach to synthesis.^[^ ^1^ ^]^

From a synthetic perspective, chemists have limited options when it comes to reaction methods. They can choose to perform a reaction solvothermally (the most widely used), photochemically, electrochemically, or mechanochemically.^[^ ^2^ ^]^ A mechanochemical reaction is a process that involves the application of mechanical energy, typically through milling or grinding.^[^ ^3^ ^]^ This approach offers several advantages over traditional methods. It provides a high yield in a shorter reaction time, ease of operation, low energy consumption, and cost efficiency, eliminates the need for bulk solvents but only minimal solvent can be utilized. In addition to these benefits, poorly soluble materials that are barely reactive under solution‐based conditions can be used as substrates. Conversely, the utilization of mechanochemistry may also entail certain drawbacks, including: presence of impurities, difficult control of particle size, and poor crystallinity. IUPAC has identified mechanochemistry as one of the top 10 innovative methodologies with the potential to transform the global landscape in terms of sustainable development.^[^ ^4^ ^]^ To the aim of developing more efficient and environmentally friendly synthetic protocols, several metrics to assess the greenness of any given chemical process (e.g., atom economy, Effective mass yield [EMY], E‐factor, etc) have been developed in accordance of the 12 principles of Green Chemistry.^[^ ^5^ ^]^ Mechanochemical approach has been employed in the synthesis of organic compounds, supramolecules, nanomaterials, MOFs and in catalysis.^[^ ^6^, ^7^, ^8^, ^9^, ^10^, ^11^, ^12^ ^]^ However, it should be noted that this field of research is still in its infancy.

Bipyridine consists of two pyridyl rings linked by a C─C bond. The most common and well‐known isomer is 2,2′‐bipyridine.^[^ ^13^ ^]^ This isomer is a chelating ligand (it coordinates generally with both N atoms) and has a rigid structure. It has been extensively researched for over a several decades, especially in the areas of coordination chemistry and catalysis. It is considered to be the most widely used ligand.^[^ ^14^, ^15^, ^16^ ^]^

On the other hand, ruthenium is a comparatively inexpensive alternative to other metals used in organometallic catalysis, such as rhodium and iridium. It has been extensively studied for a variety of applications, including transfer hydrogenation^[^ ^17^, ^18^, ^19^, ^20^, ^21^ ^]^ and metathesis reactions.^[^ ^22^, ^23^, ^24^ ^]^ Similarly, platinum and palladium complexes with dinitrogen ligands have been reported for their medicinal and catalytic activities.^[^ ^25^, ^26^, ^27^, ^28^ ^]^ Furthermore, iridium complexes, such as Cp*Ir(NN)^+^ and pentamethylcyclopentadienyl iridium have been explored for their potential in water oxidation catalysis,^[^ ^29^ ^]^ medicine,^[^ ^30^, ^31^ ^]^ photocatalysis,^[^ ^32^, ^33^ ^]^ H_2_ production,^[^ ^34^ ^]^ and water gas shift reactions.^[^ ^35^ ^]^

Concerning Iron and Cobalt, the relative tris(2,2′‐bipyridine) complexes are studied for possible application in nonaqueous redox flow batteries,^[^ ^36^ ^]^ as photoredox catalysis,^[^ ^37^ ^]^ as redox mediators in Dye‐Sensitized Solar Cells (DSSCs),^[^ ^38^ ^]^ and as complexes that were able to specifically induce collateral sensitivity in taxol‐resistant and p53‐deficient cancer cells.^[^ ^39^ ^]^

Notwithstanding the importance of the synthesis of bipyridine‐metal compounds for several decades and recent advancements in the mechanochemical synthesis of organometallic compounds^[^ ^40^, ^41^, ^42^, ^43^, ^44^, ^45^ ^]^ the development of a mechanochemical protocol for the synthesis of metal complexes bearing bipyridine remains unexplored.^[^ ^46^ ^]^

In this study, we extensively examined the reaction of more than 10 metallic precursors of Ru, Ir, Pt, Pd, Fe, and Co with 2,2′‐bipyridine in different stoichiometric ratio via mechanochemistry trying to understand the influence of the time and frequency of milling. Hence, a new green synthetic route has been explored that was found to be rapid, effective, and efficient for most of the precursors and for different metal/2,2′‐bipyridine ratio. It does not require bulk solvents or even small amounts of liquid for purification and all synthetic operations can be performed under ambient conditions and normally without air‐sensitive procedure. In order to further compare mechanochemistry with other solution‐based approaches, a comparison of yield, time of reaction, temperature for solution approach, and frequency for mechanochemistry approach, common green matrices, such us E‐factor and EMY of conventional methodologies and mechanochemical synthesis was provided^[^ ^47^ ^]^

## 2 Results and Discussion

### 2.1 Mechanochemical Synthesis of Ruthenium Bipyridine Complexes

Five common ruthenium precursors which are well explored in literature for various synthetic procedures, that is, [(p‐cymene)RuCl_2_]_2_,^[^ ^48^, ^49^ ^]^ [Ru(DMSO)_4_Cl_2_,^[^ ^50^, ^51^, ^52^ ^]^, [(CO)_3_RuCl_2_]_2_,^[^ ^53^, ^54^, ^61^ ^]^ [RuCl_2_(PPh_3_)_3_],^[^ ^55^, ^56^ ^]^ and [Ru[COD]Cl_2_]_n_^[^ 57 ^,^ ^58^ ^]^, are used as a starting point of our studies.

**Figure 1:**
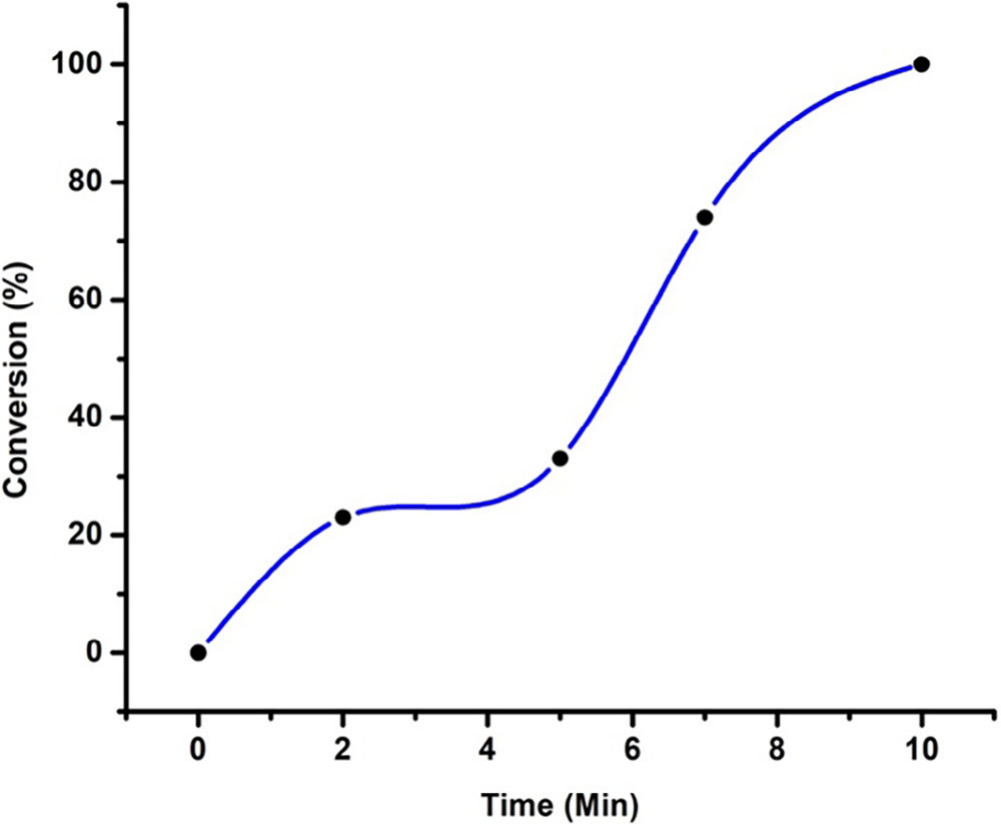
Formation of [Ru(p‐cymene)(BiPy)Cl]Cl (**1**) with milling time.

**Scheme 1:**
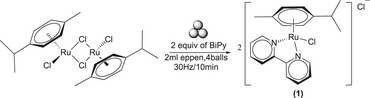
Formation of [Ru(p‐cymene)(BiPy)Cl]Cl (**1**) from [(p‐cymene)RuCl_2_]_2._

First of all, we report the mechanochemical synthesis of [Ru(p‐cymene)(BiPy)Cl]Cl **(1)** (BiPy = 2,2′‐bipyridine) from [(p‐cymene)RuCl_2_]_2_ (Figure 1). Solution‐ based cleavage of [(p‐cymene)RuCl_2_]_2_ dimer was studied in detail by several research group that report the overall yield of 71% overnight.^[^ ^59^, ^60^, ^61^ ^]^ However, we successfully synthesized **(1)** at 30 Hz milling frequency in 10 minutes (Scheme 1). Also decreasing the frequency to 20 Hz the complete conversion is achieved in the same time. The product immediately after milling was taken without washing and/or purification and then analyzed with the help of ^1^H‐NMR and ^13^C‐NMR and elemental analysis (see Experimental Section). NMR characterization and elemental analysis are in good agreement with those present in literature.^[^ ^57^ ^]^

Although we had found the optimized conditions for the synthesis of **(1)** with 100% yield, in order to understand the behavior of mechanochemical synthesis respect to the ratio of the mass of the reactants and number of balls, we doubled the mass of both reagents inside the Eppendorf. The reaction was incomplete (82% of conversion at the above best condition (20 Hz/10 minutes)). This behavior can be attributed to the increase of mass inside the Eppendorf, hindering the transfer of mechanical energy from the balls to the reactants. Therefore, less amount of energy is dissipated from the vibration of balls to all the reactants, requiring more Hz or time to complete the reaction. To confirm that the reaction takes place in the solid phase only during the milling, the NMR tube containing both the reactants in the stoichiometric amounts was analyzed immediately and after of 6 hours, showing that the reaction does not exceed 20% of conversion. Also, we conducted milling trails with smaller time, 2 minutes and 5 minutes (Figure 1). We observe 23% conversion in 2 minutes and 33% conversion in 5 minutes, providing us firm evidence that reaction taking place in the milling container (see figure S5 of Supporting Information). Furthermore, high conversion was observed at the last half of the milling time, that can be attributed to slight increase in the temperature of the container that is sufficient enough to boost the reactivity of these highly reactive precursors. Similar behavior was also observed for Ru(COD)(BiPy)Cl_2_ (see below Figure 3). In addition, we also conducted some trails with premilling of the reaction containers and balls (10 minutes at 30 Hz) in order to evaluate temperature acceleration of the reaction. However, no significant change was observed, and still complete reaction time of 10 minutes was necessary (for details see Table S1 of Supporting Information).

Taking in consideration that the ratio of the mass of the product/number of balls is important, we decided to scale‐up the synthesis of **(1)** optimizing the condition for the synthesis of 0.300 g. The suitable amount of the reagents is placed in 25 ml of Zirconia jar with 25 Zirconia balls and milled at 30 Hz for 1 hour giving complete conversion.

Next, we tried to synthesize [Ru(BiPy)(DMSO)_2_Cl_2_] **(2)** from [Ru(DMSO)_4_Cl_2_] with the same milling setup.

RuCl_2_(DMSO)_4_ + BiPy→RuCl_2_(Bipy)(DMSO)_2_ + 2DMSO

We observed that the reaction was completed in 30 minutes at 30 Hz (absence of the free BiPy signals in the ^1^H‐NMR spectrum). Furthermore, ^1^H‐NMR spectrum shows three different sets of BiPy signals coordinated to ruthenium, indicating that the selectivity is poor and that depends on the time and intensity of milling. To remove free DMSO formed during the reaction, several approaches including washing and vacuum were applied, but we were unable to isolate the dry product mixture.

We also tried milling with three equivalents of BiPy to get [Ru(BiPy)_3_]Cl_2_, but we observed only partial conversion. Other trails with high milling times were also conducted (Table S2 in Supporting Information), that further increase the number of the products and free DMSO still remains in the system.

**Scheme 2:**
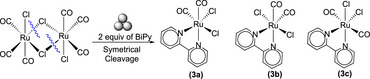
Symmetric cleavage of [(CO)_3_RuCl_2_]_2_ and formation of neutral products (3a, 3b, 3c).

**Scheme 3:**
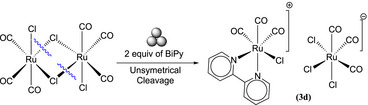
Formation of Ion pair (**3d**) due to asymmetric cleavage of [(CO)_3_RuCl_2_]_2._

The third ruthenium precursor that we used was Tricarbonyldichlororuthenium(II) dimer [(CO)_3_RuCl_2_]_2_. The reactants proved to be quite reactive (as indicated in the literature^[^ ^62^ ^]^) and we observed complete conversion in 30 Hz/30 minutes yielding both neutral and ionic products (Schemes 2 and 3).

To the best of our knowledge, the literature reports only the formation of three products from the reaction of Tricarbonyldichlororuthenium(II) dimer and Bipy, from which two of them, *cis*(Cl),*cis*(CO)‐[Ru(BiPy)Cl_2_(CO)_2_] **(3a)**, *trans*(Cl),*cis*(CO)‐[Ru(BiPy)Cl_2_(CO)_2_] **(3b)** are formed via solution synthesis^[^ ^63^ ^]^ and ionic **(3d)** in dry conditions with mortar and pestle^[^ ^58^, ^64^, ^65^ ^]^ (Figures 3 and 4). However, we observed another product (indicated as **3c**), supposed to be neutral (the position of BiPy signals in ^1^H NMR spectrum was very close to those of **3b**). In consideration of the possibilities and the execution of multiple trials for the observation of these isomers, we believe that the other product **(3c)** is *cis*(Cl),*trans*(CO)‐[Ru(BiPy)Cl_2_(CO)_2_] (Figure 3). On the other hand, the ionic products were formed due to the unsymmetric cleavage of the dimer, resulting in the ion pair **(3d)** (Scheme 3).

**Figure 2:**
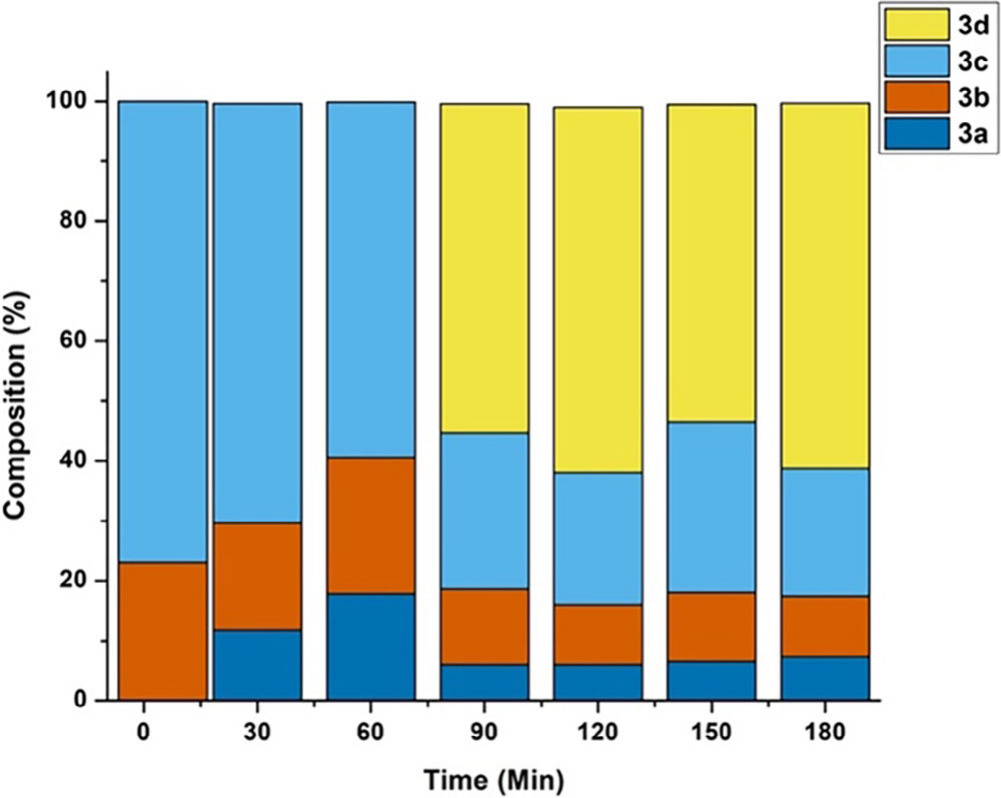
Interconversion of isomers with milling time.

In accordance with the findings reported in the literature, the milling setup yielded ionic product as the predominant when the milling process was conducted at elevated times and/or frequencies. However, significant quantities of the other neutral isomers **(3a, 3b, 3c)** were also detected in all milling procedures. The presence of ionic products was found to increase with the application of high‐energy conditions and with more time. A series of trials were conducted, varying the milling time while maintaining stoichiometric amounts of BiPy, even at low time to understand the evolution of the conversion of products into each other. A time range from 10 minutes to 3 hours with milling frequency of 10 Hz was selected and the results obtained are presented in Figure 2 and in Table S3 of the Supporting Information. Despite the yield being low at 10 Hz, the objective of these experiments was to comprehend the interconversion of the isomers. To circumvent decomposition over an extended period, low‐energy milling was selected as the milling method. The predominant product observed within 60 minutes was **(3c)**, while after this time, the ionic product was present in high quantity and remained so until 3 hours (Figure 2). This behavior of the products can be related to the performance of precursors observed in literature using a mortar and pestle,^[^ ^61^ ^]^ which clearly provides more energy than 10 Hz. Hence when subjected to high energy or extended duration, the formation of ionic products occurs. Notwithstanding, the isolation of these products in their pure form remains challenging and it is outside the scope of the work.

In order to verify that the reaction occurs exclusively during the milling process, an NMR tube containing both reactants in stoichiometric amounts was analyzed. As illustrated in Table S3 of the Supporting Information, a negligible conversion into all three neutral isomers was observed in the NMR tube. However, with time, this conversion increased, yet the reaction could not be completed in the NMR tube, as a substantial amount of free BiPy remained after a considerable period. In order to observe the conversion of one product to another within the tube, the tube was analyzed after a period of three days, but no change was observed.

**Scheme 4:**
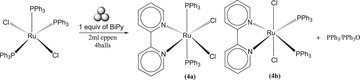
Reaction of [RuCl_2_(PPh_3_)_3_] with BiPy resulting in the formation of (**4a**) and (**4b**).

The mechanochemical process that required the greatest effort was the use of dichloro tris (triphenylphosphine)ruthenium(II) [RuCl_2_(PPh_3_)_3_] precursor. When this ruthenium precursor was used with BiPy in a 1:1 ratio under mechanochemical conditions, two distinct isomers were observed (as indicated by ^31^P‐NMR and ^1^H‐NMR, see experimental part). One is *trans*(PPh_3_)‐*cis*(Cl)[RuCl_2_(PPh_3_)_2_(BiPy)] and the other is *cis*(PPh_3_)‐*trans*(Cl)[RuCl_2_(PPh_3_)_2_(BiPy)] indicated as **(4a)** and **(4b),** respectively (Scheme 4). Solution synthesis of **(4a)** has been reported by various groups that shows the possible chemical shift around *δ* = 20 ppm ^31^P{^1^H}‐NMR depending on the solvent used.^[^ ^66^, ^67^, ^68^ ^]^ However, we observed the presence of another distinct product **(4b)** with chemical shift of *δ* = 54 ppm in the ^31^P{^1^H}‐NMR spectra record in CDCl_3_. When the mixture is analyzed with ^1^H‐NMR, it also aligns with the same argument. It is noteworthy that *cis*(PPh_3_)‐*trans*(Cl)[RuCl_2_(PPh_3_)_2_(BiPy)] has not been previously reported in the literature. According to Batista and colleagues, when two PPh_3_ is present at the trans position in different neutral ruthenium complexes bearing nitrogen and halogen ligands, the possible chemical shift is the same expected for isomer **(4a)**. Within the same study, it is highlighted that when the N atom of the nitrogen ligand is present trans to the phosphorus of PPh_3_, a higher chemical shift around 50 ppm should be expected, as observed for **(4b)**.^[^ ^64^ ^]^

In the course of optimizing the synthesis of both complexes, the trials were divided into two sub‐trials at 20 Hz and 30 Hz, respectively, over variable time periods (see Table S4 of the Supporting Information for details). The targeted synthesis of isomer **(4a)** was performed at 20 Hz, and it was observed that no product appeared until the first hour of milling. However, after 90 minutes, the desired isomer **(4a)** was observed, accompanied by PPh_3_, phosphine oxide (PPh_3_O), and the starting Ru precursor. In order to isolate pure **(4a)**, a washing process with pentane was implemented. This method effectively removed the Ru precursor, PPh_3,_ and BiPy. Nevertheless, repeated washings were unable to fully eliminate PPh_3_O. In an effort to mitigate the presence of PPh_3_O, an argon flux was employed to remove air from the eppendorf, which was then sealed tightly. This approach resulted in a substantial reduction in the incorporation of PPh_3_O.

In addition to milling at 20 Hz, milling of the reactants in the same composition was also conducted at 30 Hz. The appearance of isomer **(4a)** was observed to be rapid, occurring within five minutes, accompanied by the presence of PPh_3_O and the free Ru precursor. Following these procedures, the presence of isomer **(4b)** was noted after 10 minutes. A series of additional trials was conducted, which underlined the conversion of isomer **(4a)** to isomer **(4b)** under more energetically demanding conditions. To understand the conversion of isomer **(4a)** to isomer **(4b)**, it is crucial to refer to the trails in Table S4 of Supporting Information, which indicate that isomer **(4a)** undergoes conversion (as well as decomposition) to isomer **(4b)**. Furthermore, milling for an extended period (3–4 hours) at 20 Hz was conducted, that further indicates the decomposition of the products to starting ruthenium precursor. Subsequent washing with pentane removed both the starting material, BiPy and the minute amount of PPh_3_O. With this procedures, mixture of products contained a concentration of 8% of isomer **(4b)** (see Supporting Information) is obtained. Additionally, a few trials were conducted with a 25 ml zirconia jar. In both cases, despite the low concentration of PPh_3_O, the high contact energy in the larger jar and ball resulted in the observation of high concentrations of isomer **(4b)** even at low times, with both 20 and 30 Hz. During the process of removing PPh_3_O from the system, the samples were washed with diethyl ether. Subsequently, both the washed and unwashed samples were subjected to NMR analysis. It was observed that the amount of products present in the spectra increased in the presence of washing, suggesting that diethyl ether may accelerate the reaction at the interface.

**Scheme 5:**
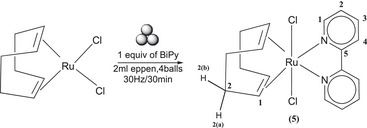
Synthesis of Ru(COD)(BiPy)Cl_2_ (**5**) from [Ru(COD)Cl_2_]_n_ polymer.

The complex **(5)** (Scheme 5) in the series of Ru‐BiPy‐based compounds was synthesized from [Ru[COD]Cl_2_]_n_ polymer with one equivalent of BiPy. The complete conversion of the reactants into the target **(5)** compound was achieved through a mechanochemical approach, which effectively break down the polymer at 30 Hz for 30 minutes.

**Figure 3:**
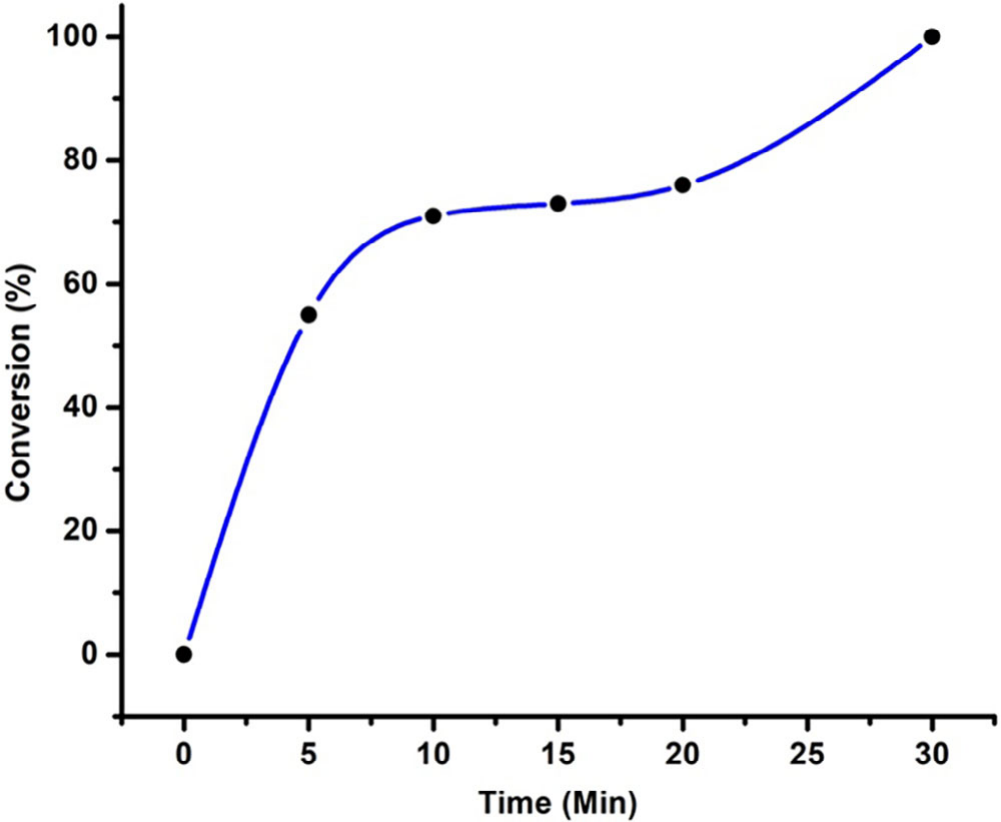
Formation of Ru(COD)(BiPy)Cl_2_ (**5**) with time (30 Hz).

The complete characterization of **(5)** was conducted through multinuclear NMR and elemental analysis, revealing its remarkable stability and purity, with no decomposition observed even after 90 minutes of milling. The formation of **(5)** over milling time was also studied (see Figure 3). The trend in Figure 3 indicates that the reaction was very fast at the beginning and that more than 50% conversion was achieved within the first five minutes (for details see Table S5 of Supporting Information). Furthermore, attempts were made to synthesize Ru(BiPy)_3_Cl_2_^[^ ^69^, ^70^ ^]^ and Ru(BiPy)_2_Cl_2_^[^ ^71^ ^]^ in mechanochemical conditions with the appropriate stoichiometric addition of dinitrogen ligand, but Ru(COD)(BiPy)Cl_2_ remained coordinate to the ruthenium center. This outcome is of significant interest, given that the compounds obtained in solution by reacting the polymer and one equivalent of bipyridine are compounds Ru(BiPy)_3_Cl_2_ and Ru(BiPy)_2_Cl_2_. To the best of our knowledge, **(5)** has not observed in the literature, thus demonstrating that the mechanochemical approach can indeed complementary to solution approach and, also that **(5)** can be used as an intermediate and starting point for the synthesis of other compounds.

### 2.2 Mechanochemical Synthesis of Pd‐BiPy and Pt‐BiPy‐ Based Complexes

**Scheme 6:**
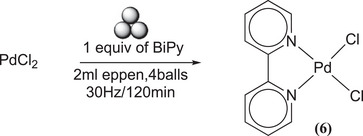
Synthesis of Pd(BiPy)Cl_2_ (**6**).

The mechanochemical synthesis of Pd(BiPy)Cl_2_ **(6)** was found to be a relatively straightforward and expeditious process when employing ball milling. To this end, a series of trials was conducted, utilizing a 1:1 ratio of both reactants at varying time intervals ranging from 5 minutes to 2 hours and at frequencies ranging from 15 Hz to 30 Hz (Scheme 6). The reactants were found to be reactive, with an approximate yield of 83% obtained at 15 Hz and 5 minutes. The yield increased with milling time, and the reaction was complete after 120 minutes. Consequently, it can be deduced that, for this particular complex, mechanochemistry provides two distinct options: a high yield (100% without purification and washing) is obtained with 2 hours of milling, or a good yield (85%) is obtained with low time (5 minutes).

**Scheme 7:**
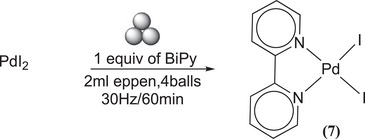
Synthesis of Pd(BiPy)I _2_ (**7**).

In consideration of the behavior of PdCl_2_, PdI_2_ (scheme 7) was also milled with BiPy to obtain Pd(BiPy)I_2_ **(7)**. The reaction was observed to be faster than when PdCl₂ is used, with complete conversion being achieved within one hour (see Table S7 of the Supporting Information for details). A comparison between the mechanochemical method and the solution method is of particular interest. Indeed, complexes **(6)** and **(7)** are obtained in solution by adding 10 mL of methanol for every 200 mg of palladium precursors and reacting for 4/6 hours. Furthermore, precipitation, washing with more methanol and drying are required.^[^ ^69^ ^]^

**Scheme 8:**
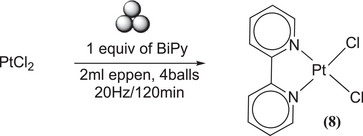
Synthesis of Pt(BiPy)Cl _2_ (**8**).

In a similar manner, efforts were made to synthesize Pt(BiPy)Cl_2_ **(8)**, Scheme 8. However, in this instance, a very low conversion was observed at a low time (15 Hz/5 minutes). Subsequent trials were conducted (see Table S8 of Supporting Information) by subjecting the samples to more energetic conditions to achieve complete conversion. Notwithstanding, the ^1^H‐NMR analysis of the samples revealed the presence of two distinct products. The predominant product, accounting for approximately 70% of the total, was identified as the targeted compound **(8)**, as reported by various research groups employing solution synthesis methods.^[^ ^25^, ^72^ ^]^ Notwithstanding the attainment of complete conversion at 20 Hz and 120 minutes, the concentration of the other product, in conjunction with the targeted main product, remains constant (approximately 30%). The process of washing was conducted with the objective of removing the other product; however, this approach did not yield the desired outcome. Nevertheless, it has been observed that the process does effectively remove any excess BiPy when such is present. In relation to the characterization of the minority product, it can be hypothesized that both nitrogen of bipyridine are platinum‐coordinated, based on the multinuclear characterization conducted using nuclear magnetic resonance (NMR). This suggests a dimeric ionic structure of the type [Pt(BiPy)(Cl_2_)Pt(BiPy)]Cl_2_.

### 2.3 Mechanochemical Synthesis of Iron and Cobalt Bipyridine Complexes

**Scheme 9:**
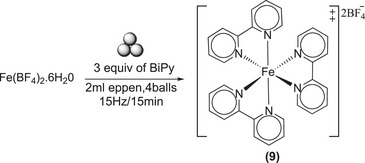
Synthesis of Fe(BiPy)_3_(BF_4_)_2_ (**9**).

In this section of the study, the Fe(BF_4_)_2_.6H_2_O precursor was utilized to synthesize Fe(BiPy)_3_(BF_4_)_2_ **(9)**, which exhibited a notable reaction via a mechanochemical approach (see Scheme 9). The precursor demonstrated a high degree of reactivity with BiPy, and the reaction was completed within 15 minutes at 15 Hz. Similar outcomes were observed under high‐energy conditions (30 minutes at 30 Hz). However, when a double amount of both reactants was used in the same Eppendorf and with the same number of balls, the conversion was found to be a little low (80%), which can be attributed to the low transfer of energy from the balls to the reactants, as seen before for other trials. Also in this case, comparison between the mechanochemical method and the solution method is of particular interest. In fact, complex **(9)** is obtained in solution by adding 10 mL of ethanol for about 20 mg of iron precursors and reacting for 3 hours. Furthermore, **(9)** was collected via filtration and washed with ethanol and dried in a vacuum oven overnight.^[^ ^71^ ^]^

**Scheme 10:**
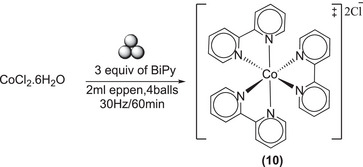
Synthesis of Co(BiPy)_3_Cl_2_ (**10**).

Similar type of strategy was used for the synthesis of Co(Bipy)_3_Cl_2_ **(10)** starting from CoCl_2_.6H_2_O and three equivalents of bipyridine (scheme 10). The reaction was quick, and complete conversion was achieved after 60 minutes. As the resultant product contain paramagnetic Co(II), therefore formation of [Co(BiPy)_3_]^++^ was confirmed from FTIR‐ATR, and mass spectrometry (see Supporting Information for details).

### 2.4 Mechanochemical Synthesis of Ir‐BiPy Complex

**Scheme 11:**
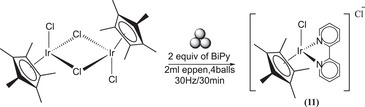
Synthesis of [Ir(Cp*)(BiPy)Cl]Cl (**11**).

Pentamethylcyclopentadienyl Iridium dichloride dimer [IrCp*Cl_2_]_2_ was milled with BiPy ligand to form the following complex [Ir(Cp*)(BiPy)Cl]Cl **(11)**, Scheme 11.

The dimer is prone to a rapid reaction in ball milling conditions, forming the targeted product **(11)**. The milling process was conducted under two distinct conditions: 20 Hz for 20 minutes and 30 Hz for 30 minutes. From NMR measurements we can conclude that the reaction was complete at 30 Hz in 30 minutes and no purification is required. However, at 20 Hz in 20 minutes, the reaction was incomplete, yielding approximately 80% of conversion of the desired product. Also in this case, comparison between the mechanochemical method and the solution method reveals that complex **(11)** is obtained in solution by adding 30 mL of ethanol for about 100 mg of iridium dimer and reacting for 5 hours at 50° C. Furthermore, **(11)** was collected via filtration and washed with pentane and dried in a vacuum oven overnight.^[^ ^72^ ^]^

In conclusion, the conversion of most precursors has been accomplished, with a time span ranging from 10 minutes to 2 hours. In general, a lower level of complexity is exhibited compared to the solution approach and precipitation, washing, and dryness are not necessary. However, selectivity of the synthesis of certain products has been observed to present challenges. Further details pertaining to the trials conducted with optimal milling conditions for all precursors can be found and summarized in Table 1.

**Table 1:** Milling Condition for all the precursors used in this work

Precursor	Molar Ratio of BiPy	Milling Freq	Time [min]	Conv [%]	Product
[(p‐cymene)RuCl_2_]_2_	1:2	30	10	99	[Ru(p‐cymene)(BiPy)Cl]Cl **(1)**
[Ru(DMSO)_4_Cl_2_]	1:1	30	30	99	Multiple Products
[(CO)_3_RuCl_2_]_2_	1:2	30	30	99	[Ru(BiPy)Cl_2_(CO)_2_] as **(3a, 3b, 3c, and 3d)** ^[^ ^a^ ^]^
[RuCl_2_(PPh_3_)_3_]	1:1	30/20	‐	‐	*Cis*(Cl)‐[RuCl_2_(PPh_3_)_2_(BiPy)] **(4a)** (42%), *Trans*(Cl)‐[RuCl_2_(PPh_3_)_2_(BiPy)] **(4b)** (8%) ^[^ ^b^ ^]^
[Ru[COD]Cl_2_]_n_	1:1	30	30	99	Ru[COD][BiPy]Cl_2_ **(5)**
PdCl_2_	1:1	30	120	99	Pd(BiPy)Cl_2_ **(6)**
PdI_2_	1:1	30	60	99	Pd(BiPy)I_2_ **(7)**
PtCl_2_	1:1	30	120	99	Pt(BiPy)Cl_2_ **(8)**
Fe(BF_4_)_2_.6H_2_O	1:3	15	15	99	Fe(BiPy)_3_(BF_4_)_2_ **(9)**
CoCl_2_.6H_2_O	1:3	30	60	99	Co(BiPy)_3_Cl_2_ **(10)**
[IrCp*Cl_2_]_2_	1:2	30	30	99	[Ir(Cp*)(BiPy)Cl]Cl **(11)**

^[a]^ See Table S3, Supporting Information for major details

^[b]^ See Table S4, Supporting Information for major details

**Table 2:** Comparison of green metrics for solution and mechanochemical synthesis

	MECHANOCHEMICAL SYNTHESIS [THIS WORK]			SOLUTION SYNTHESIS^[^ ^a^ ^]^			
COMPLEX	E‐Factor	EMY	Time [min]	E‐Factor	EMY	Time^[^ ^b^ ^]^ [min]	Temp [^o^C]	Ref.
[Ru(p‐cymene)(BiPy)Cl]Cl	0	100	10	0.4	71	240	25	[57]
[Ru(BIPY)(DMSO)_2_cL_2_]	0.32	75.6	30					
[Ru(BIPY)CL_2_(CO)_2_]	0	100	30	16.5	5.68	60	RT	[61]
[RuCL_2_(PPh_3_)_2_(BiPy)]	534	0.18	30	569	0.17	45	RT	[68]
Ru[COD][BiPy]Cl_2_	0	100	30					
Pd(BiPy)Cl_2_	0	100	120	18.1	5.3	240	RT	[73]
Pd(BiPy)I_2_	0	100	60					
Pt(BiPy)Cl_2_	0	100	120	8.2	10.7	720	60	[25]
Fe(BiPy)_3_(BF_4_)_2_	0	100	15			180	RT	[74]
Co(BiPy)_3_Cl_2_	0	100	60	‐	‐	360	RT	[75]
[Ir(Cp*)(BiPy)Cl]Cl	0	100	30	162	0.6	300	50	[76]

^[a]^ These values are calculated according to the procedure available in literature (with missing amounts of some solvents, that may further increase the amount of waste produced). For details of calculation check Supporting Information.

^[b]^ This time only includes the time of reaction for solution synthesis (time of workup or time for purification is not included)

### 2.5 Green Metrics: Solution‐ Versus Mechano‐ Synthesis

As outlined in the previous section, the decision to eliminate the use of bulk solvent during the reaction process will undoubtedly contribute to a more sustainable procedure, given that it eliminates the generation of waste. In this context, in contrast to conventional solution‐based chemistry, where reactions frequently necessitate the use of excess reagents to achieve complete conversion, mechanochemical reactions can often be conducted with an equimolar amount of reagents. This efficiency can be attributed to the effective mixing of reagents caused by the milling action, which enhances reagent reactivity, ensuring the complete utilization of the initial materials. This contributes to the minimization of excess reagents, leading to a reduction in waste generation and a simplification of product purification.

As there is no single parameter that can definitively indicate whether a chemical reaction is entirely green or sustainable, in order to consolidate the mechanochemical synthetic approach as a green method, a comparison was made between our synthesis and those found in the existing literature. Both (mechanochemical and solution) of the approaches were analyzed using two metrics, the E‐factor [E‐Factor = (Mass of Waste)/(Mass of Product)] and the EMY [EMY = (Mass of Product)/(Total Mass of Material Used) × 100], which are instrumental in analyzing the environmental effectiveness of the methodology. The E‐factor and EMY have been found to be useful in determining the amount of waste produced during a chemical process, but we would like to highlight at this stage that solution reports don't provide complete experimental details required for the calculation of green metrics: especially the solvents request for crystallization and purification. The decision was taken to calculate the values solely with the masses of the reagents, products, byproducts, and solvent utilized for the synthesis, excluding the purification.

It is reported that the optimal values for most of the synthesized products (**1**, **2**, **3**, **5**, **6**, **7**, **8**, **9**, **10**, **11**) have been found to be E‐factor ≅ 0 and EMY ≅ 100 (Table 2, for details see section 3 of Supporting Information). However, in solution conditions, as reported in the literature, these values were found to be considerably higher (for E‐factor) and lower (for EMY). This outcome unequivocally demonstrates that the present mechanochemical process is significantly more sustainable than the solution process and will undoubtedly be further developed in the future.

**Figure 4:**
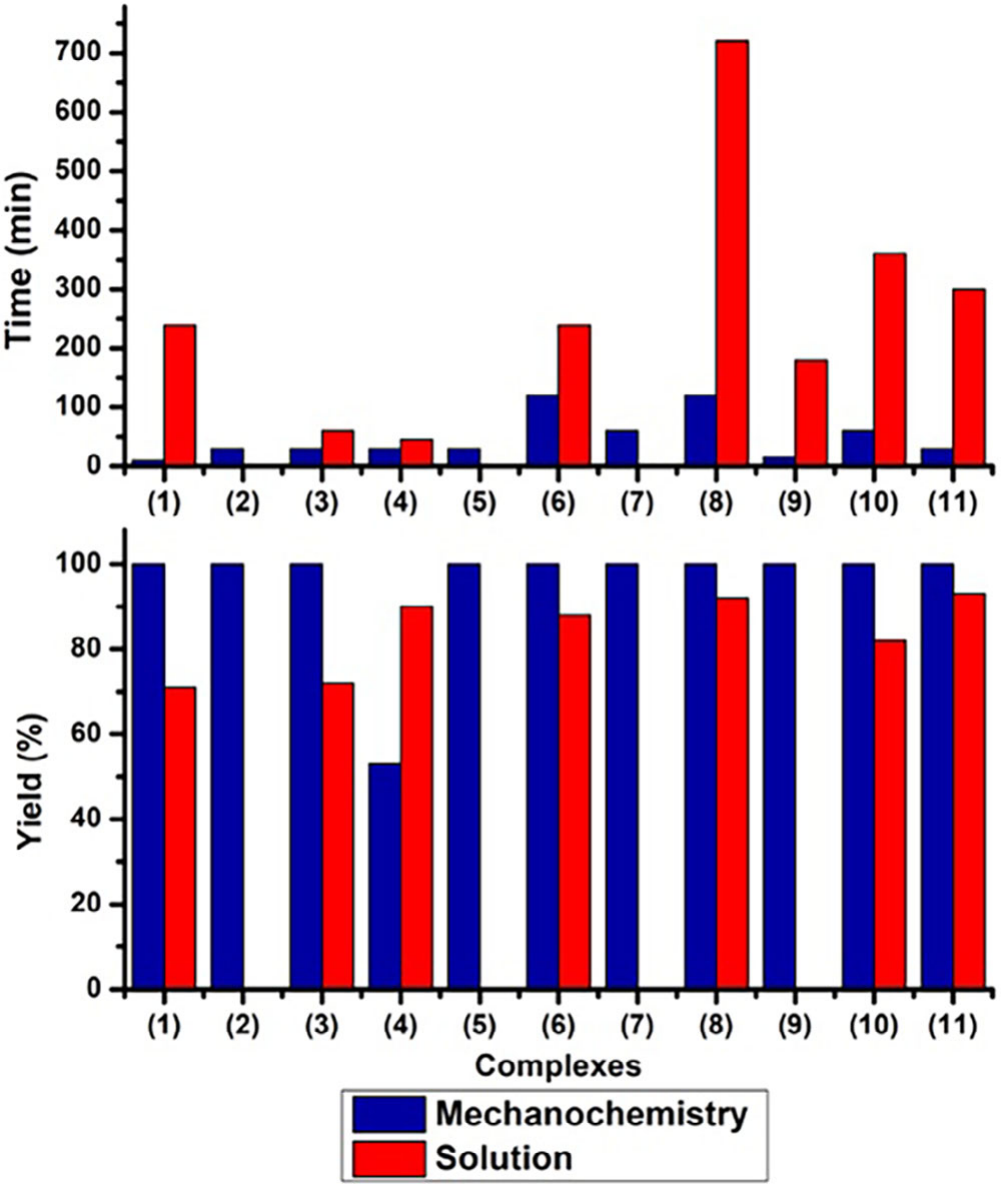
Comparison of Solution‐ and Mechanosynthesis.

In addition to the green factors (e.g., E‐factor and EMY), mechanochemical synthesis has been shown to be advantageous in terms of high yield and low reaction time (see Figure 4). A comparison of the yield for the precursors reported in this study reveals that solution synthesis has never reported a complete 100% yield (mainly due to the use of bulk solvents and purification steps). However, this approach appears to be feasible (though not universally) for mechanochemical reactions with very low reaction times. It is evident that mechanosynthesis exhibits superiority over conversion solution‐based strategies on the grounds of its capacity to produce a minimal environmental impact, in addition to its propensity for expeditious and effective reactions.

## 3 Conclusion

In conclusion, a simple mechanochemical protocol was demonstrated that enables highly efficient and rapid solid‐state synthesis of metal bipyridine complexes without the use of bulk solvents, higher temperature, longer reaction time, or a complicated reaction setup involving inert gases.

By accurately adjusting the BiPy/metal precursor ratio, the milling time and frequency it is possible to ascertain the optimal conditions for the metal precursors utilized. Notably, most metal‐bipy complexes were obtained within 30/60 minutes in good to high yields and in most of the cases the conversion is quantitative and no purification is necessary (or at least simple washing of slightly excess of BiPy).

A gram‐scale reaction under mechanochemical conditions was also demonstrated for compound Ru(p‐cym)(BiPy)Cl_2_^[^ ^1^ ^]^ and Co(BiPy)_3_Cl_2_^[^ ^10^ ^]^ (for details see Tables S1 and S10 of Supporting Information). In addition, compound Ru[COD][BiPy]Cl_2_^[^ ^5^ ^]^ was obtained for the first time under these conditions; in fact, the classical route in solution from precursor [Ru[COD]Cl_2_]_n_ only yielded compounds Ru[BiPy]_2_Cl_2_ and Ru[BiPy]_3_Cl_2_ suggesting that mechanochemistry approach can be complementary to common solvothermally approach.

In order to provide a more comprehensive comparison of mechanochemistry with other solution‐based approaches, a range of metrics was analyzed for the synthetized and isolated complexes. Yield, reaction time, temperature for the solution approach and frequency for the mechanochemical approach, together with two common green matrices, such as the E‐factor and the EMY of conventional methodologies and mechanochemical synthesis, were considered. A comparison of the parameters clearly indicates (Table 1) that the mechanochemical approach is more sustainable, expeditious, and energy‐efficient in the synthesis of compounds.

These findings imply that the present mechanochemistry method has the potential for wide application as an efficient, cost‐effective, and sustainable synthesis technique for organometallic compounds and it is reasonable to predict that rapid development will be observed in the coming years. Further studies have been initiated in our laboratory, with a focus on various frequently used ligands, including carbenes and phosphines. These investigations will form the basis of subsequent research activities.

## 4 Experimental Section

[(p‐cymene)RuCl_2_]_2_ and [RuCl_2_(PPh_3_)_3_] were purchase from Johnson Matthey. [(CO)_3_RuCl_2_]_2_ and PtCl_2_ were purchase from Alfa Aesar. FeBF_4_.6H_2_O was purchase from Sigma Aldrich. PdCl_2_, PdI_2_ and PtCl_2_ were purchase from Carlo Erba. 2,2′‐Bipyridine was purchase from Riedel‐de Haen. [Ru(DMSO)_4_Cl_2_], [Ru[COD]Cl_2_]_n_, and [IrCp*Cl_2_]_2_ were purchase from Strem Chemicals, Inc. Elemental analysis (C, H, N) was carried out with a Carlo Erba 1106 elemental analyzer. The ^1^H, ^13^C, and ^31^P NMR spectra were acquired using a Bruker Avance III HD 400 MHz spectrometer equipped with a broadband 5 mm probe (1H/BBF iProbe) with a z‐axis gradient (50 G/cm). Chemical shifts are reported in parts per million and calibrated to the solvent residue.

Mechanochemical synthesis of all Metal‐Bipyridine complexes was carried out using Retsch Ball Mill (MM 500 Vario) with 2 ml Eppendorf and zirconia balls (3 mm/∼0.4 g) or in zirconia jar of 25 ml with zirconia ball (15 mm/∼11.4 g). In Eppendorf, the suitable amounts of the reactants (metallic precursor and 2′2‐Bipyridne) were taken with four zirconia balls inside. In order to calculate the final mass of the products, the Eppendorf (or zirconia Jar) and balls were appropriately calibrated. The Eppendorf is then closed (under Argon when required to avoid oxidation by air), air tightened with the help of parafilm and then milled at suitable milling conditions (as highlighted below for each synthesis). Approximately 5 mg of the solid sample is extracted from Eppendorf (or jar) and dissolved in a suitable deuterated solvent for NMR analysis. A second portion is then utilized for elemental analysis. The syntheses were replicated three times, except for the 300 g^[^ ^1^ ^]^ and 1000 g^[^ ^10^ ^]^ scale tests.

### MC synthesis of [Ru(p‐cymene)(BiPy)Cl]Cl^[^ ^1^ ^]^

[(p‐cymene) RuCl_2_]_2_ (15 mg, 0.0244 mmol) and BiPy (7.65 mg, 0.049 mmol) are milled at 30 Hz for 10 minutes. ^1^H‐NMR spectrum in CDCl_3_: *δ* = 9.7 (d, J = 4.5 Hz, 2H), 8.38 (d, J = 7.8, 2H), 8.1 (t, J = 7.62, 2H), 7.75 (t, J = 5.8 Hz, 2H), 6.2 (d, J = 4.57, 2H), 6.1(d, J = 4.9 Hz, 2H), 2.72(m, 1H), 2.3(s, 3H), 1.08(d, J = 6.82 Hz, 6H) (Figure S3). ^13^C‐NMR spectrum in CDCl_3_: *δ* = 156.5 (C─H bipyridine), 154.48 (quaternary C of bipyridine), 139.5 (C─H of bipyridine), 128.1 (C─H of bipyridine), 123.5 (C─H of bipyridine), 104.9 (quaternary C of p‐cymene), 104.2 (quaternary C of p‐cymene), 87.3 (C─H of p‐cymene), 84.6 (C─H of p‐cymene), 31.2 (CH_3_ of cymene), 22.2 (CH_3_ of isopropyl), 19.1 (C─H of isopropyl) (Figure S4). Elemental Analysis: calculated (%) for (C_21_ H_25_Cl_2_N_2_Ru): C, 52.78; H, 5.31; Cl, 14.90; N, 5.82; Ru, 21.19. Found (%): C, 52.68; H, 5.39; N, 5.78.^[^ ^56^ ^]^

### MC synthesis of Ru(BiPy)(DMSO)_2_Cl_2_^[^ ^2^ ^]^

15 mg of [Ru(DMSO)_4_Cl_2_] (0.31 mmol) and BiPy (4.84 mg, 0.31 mmol) are milled at 20 Hz for 20 minutes. ^1^H‐NMR spectrum in CDCl_3_: *δ* = 9.4 (d, J = 4.6 Hz, 2H), 8.64(d, J = 8.07 Hz, 2H), 8.28 (t, J = 7.55 Hz, 2H), 7.8 (t, J = 6.9 Hz 2H) (Figure S6).

### MC synthesis of Ru(BiPy)Cl_2_(CO)_2_ (3a,3b,3c,3d)

15 mg [(CO)_3_RuCl_2_]_2_ (0.029 mmol) and BiPy (9.2 mg, 0.58 mmol) are milled for 90 minutes at 10 Hz. ^1^H‐NMR spectrum in CDCl_3_: *δ* = 9.8 (d, J = 5.7 Hz, 2H), 9.38 (d, J = 6.5 Hz, 2H), 9.2 (d, J = 5.5 Hz, 2H), 9.04(d, J = 5.5 Hz, 2H), 8.88 (d, J = 5 Hz, 2H), 8.7 (d, J = 8.2 Hz, 2H), 8.55 (d, J = 8.1 Hz, 2H), 8.4(t, J = 7.88 Hz, 2H), 8.3–8.05 (m)(several peaks overlapping), 8.01 (t, J = 7.71 Hz, 2H), 7.8 (t, J = 6.7 Hz, 2H), 7.76 (t, J = 6.73 Hz, 2H), 7.7 (t, J = 6.56 Hz, 2H), Peaks overlap from 7.55–7.67, 7.49 (t, J = 6.2 Hz, 2H) (Figure S7).^[^ ^60^ ^]^

### MC Synthesis of RuCl_2_(PPh_3_)_2_(BiPy) (4a, 4b)

15 mg of [RuCl_2_(PPh_3_)_3_] (0.0156 mmol) and BiPy (2.68 mg, 0.17 mmol) are milled at 20 Hz and 30 Hz at variable time. ^1^H‐NMR spectrum in CD_2_Cl_2_: *δ* = 10.2 (d, J = 6.33 Hz, 2H), 8.94 (d, J = 5.94 Hz, 2H), 8.76 (d, J = 5.76 Hz, 2H), 8.69 (d, J = 6.2 Hz, 2H), 8.46 (d, J = 8 Hz, 2H), 8.2 (d, J = 7.9 Hz, 2H), 8.1 (d, J = 8.5, 2H), Overlapping of peaks from 7–8, 6.9 (t, J = 6.13 Hz 2H), 6.45 (t, J = 6.6 Hz, 2H) (Figure S8). ^31^P‐NMR spectrum in CD_2_Cl_2_: *δ* = 23.9(s, 2 trans P), 27.4(s, PPh_3_O), 56.08(s, 2 cis P) (Figure S9).

### MC Synthesis of Ru(COD)(BiPy)Cl_2_^[^ ^5^ ^]^

15 mg (0.054 mmol) of [Ru[COD]Cl_2_]_n_ and BiPy (8.35 mg, 0.54 mmol) were milled at 30 Hz for 30 minutes. ^1^H‐NMR spectrum in CDCl_3_: *δ* = 8.21 (d, J = 5.62 Hz, 2H_1_), 8.17(d, J = 8.18 Hz, 2H_4_), 7.97(t, J = 7.85 Hz, 2H_2_), 7.48 (t, J = 6.58 Hz, 2H_3_), 4.7(m, H_5_), 2.81(m, H_6(a)_), 2.26 (m, H_6(b)_) (Figure S10). ^13^C‐NMR spectrum in CDCl_3_: *δ* = 157.1(quaternary C of bipyridine), 150.1 (C─H of bipyridine), 138 (C─H of bipyridine), 126.3 (C─H of bipyridine), 123 (C─H of bipyridine), 91.6 (C─H of COD), 29.4 (C─H_2_ of COD) (Figure S11). Elemental Analysis: calculated (%) for (C_18_H_20_Cl_2_N_2_Ru): C, 49.57; H, 4.66; Cl, 16.23; N, 6.39; Ru, 23.15 Found (%): C, 49.47; H, 4.76; N, 6.29.

### MC Synthesis of Pd(BiPy)Cl_2_^[^ ^6^ ^]^

15 mg of PdCl_2_ (0.085 mmol) and BiPy (13.2 mg, 0.085 mmol) were milled for 2 hours at 20 Hz. ^1^H‐NMR spectrum in DMSO: *δ* = 9.14 (d, J = 5.9 Hz, 2H), 8.59 (d, J = 8.3 Hz, 2H), 8.37 (t, J = 7.8 Hz, 2H), 7.82 (t, J = 6.7 Hz, 2H) (Figure S12). ^13^C‐NMR spectrum in DMSO: *δ* = 156.9 (quaternary C of bipyridine), 150.2 (C─H of bipyridine), 141.7 (C─H of bipyridine), 127.8 (C─H of bipyridine), 124.4 (C─H of bipyridine) (Figure S13). Elemental Analysis: calculated (%) for (C_10_H_8_Cl_2_N_2_Pt): C, 28.48; H, 1.88; Cl, 16.72; N, 6.67; Pt, 46.25. Found (%): C, 28.34; H, 1.98; N, 6.57.^[^ ^72^ ^]^

### MC synthesis of Pd(BiPy)I_2_^[^ ^7^ ^]^

15 mg of the PdI_2_ (0.0416 mmol) and BiPy (6.5 mg, 0.0416 mmol) were milled for 1 hour at 30 Hz. ^1^H‐NMR spectrum in CDCl_3_: *δ* = 10.12 (d, 5.9 Hz, 2H), 8.3(t, J = 7.78 Hz, 2H), 8.08 (d, J = 7.83 Hz, 2H), 7.63(t, J = 7.26 Hz, 2H) Figure S14. ^13^C‐NMR spectrum in DMSO: 157 (quaternary C of bipyridine), 152.9 (C─H of bipyridine), 141.1 (C─H of bipyridine), 128.23 (C─H of bipyridine), 124.67 (C─H of bipyridine) Figure S15. Elemental Analysis: calculated (%) for (C_10_H_8_I_2_N_2_Pd): C, 23.28; H, 1.54; I, 49.13; N, 5.48; Pd, 20.57. Found (%): C, 23.16; H, 1.68; N, 5.34.

### MC Synthesis Pt(BiPy)Cl_2_^[^ ^8^ ^]^

15 mg of PtCl_2_ (0.056 mmol) and BiPy (8.75 mg, 0.056 mmol) were milled at 20 Hz for 60 minutes. ^1^H‐NMR spectrum in DMSO: *δ* = 9.5 (d, J = 5.88 Hz, 2H), 8.7 (d, J = 4.3 Hz, 2H), 8.59 (d, J = 8.16 Hz, 2H), 8.42 (t, J = 7.78 Hz, 2H), 7.9 (t, J = 7.68 Hz, 2H), 7.85 (t, J = 6.7 Hz, 2H), 7.5 (t, J = 6.03 Hz, 2H) (Figure S16). ^13^C‐NMR spectrum in DMSO: *δ* = 157.3 (quaternary C of bipyridine), 149.6 (C─H of bipyridine), 148.8 (C─H of bipyridine), 141 (C─H of bipyridine), 128.2 (C─H of bipyridine), 124.7 (C─H of bipyridine) (Figure S17). Elemental Analysis: calculated (%) for (C_10_H_8_I_2_N_2_Pt): C, 28.46; H, 1.89; Cl, 16.80; N, 6.62; Pt, 46.23. Found (%): C, 28.34 H, 2.01; N, 6.51.^[^ ^25^ ^]^

### MC Synthesis of Fe(BiPy)_3_(BF_4_)_2_^[^ ^9^ ^]^

15 mg of Fe(BF_4_)_2_.6H_2_O (0.04 mmol) and 20.8 mg of BiPy (0.12 mmol) were milled at 15 Hz for 15minutes. ^1^H‐NMR spectrum in CDCl_3_: *δ* = 8.87 (d, J = 8.13 Hz, 2H), 8.28 (t, J = 7.78 Hz, 2H), 7.75 (d, J = 5.53 Hz, 2H), 7.6 (t, J = 7.4 Hz, 2H) (Figure S18). ^13^C‐NMR spectrum in CDCl_2_: *δ* = 159 (qua ternary C of bipyridine), 153.9 (C─H of bipyridine), 139 (C─H of bipyridine), 127.8 (C─H of bipyridine), 123.9(C─H of bipyridine) (Figure S19). Elemental Analysis: calculated (%) for (C_30_H_24_B_2_F_8_FeN_6_): C, 51.62; H, 3.47; B, 3.10; F, 21.77; Fe, 8.00; N, 12.04 Found (%): C, 51.46; H, 3.57; N, 11.88.^[^ ^74^ ^]^

### MC Synthesis of Co(BiPy)_3_Cl_2_^[^ ^10^ ^]^

15 mg of CoCl_6_.6H_2_O (0.063 mmol) and 30.5 mg of BiPy (0.19 mmol) were milled at 30 Hz for 60 minutes *v*CN = 1596.7 cm^−1^. *m/z* = 263.5 (*z* = 2). Elemental Analysis: calculated (%) for (C_30_H_24_Cl_2_CoN_6_): C, 60.21; H, 4.04; Cl, 11.85; Co, 9.85; N, 14.04 Found (%): C, 60.04; H, 4.22; N, 13.90.^[^ ^75^ ^]^

### MC Synthesis of [Ir(Cp*)(BiPy)Cl]Cl^[^ ^11^ ^]^

10 mg of [IrCp*Cl_2_]_2_ (0.012 mmol) and 4.12 mg of BiPy (0.026 mmol) were milled for 30 minutes at 30 Hz. ^1^H‐NMR spectrum in CDCl_3_: *δ* = 9.36 (d, J = 8.05 Hz, 2H), 8.83 (d, J = 5.53, 2H), 8.29 (t, J = 7.37 Hz, 2H), 7.79 (t, J = 6.09 Hz, 2H) (Figure S21). ^13^C‐NMR spectrum in CDCl_3_: 155.6 (quaternary C of bipyridine), 150.6 (C─H of bipyridine), 141 (C─H of bipyridine), 128.7 (C─H of bipyridine), 126.5 (C─H of bipyridine), 89.2 (quaternary C of pentadienyl ring), 8.97 (C─H_3_ of pentamethyl) (Figure S22). Elemental Analysis: calculated (%) for (C_20_H_23_Cl_2_IrN_2_): C, 43.32; H, 4.18; Cl, 12.79; Ir, 34.66; N, 5.05. Found (%): C, 43.18; H, 4.24; N, 4.93.^[^ ^76^ ^]^

## Supporting Information

The acquired NMR spectra, detailed experimental procedures, and additional experimental data are included in the Supporting Information.

## Conflict of Interest

The authors declare no conflict of interest.

## Supporting information



Supporting Information

## Data Availability

The data that support the findings of this study are available in the supplementary material of this article.
