# Supplementary material for: Rapid and Efficient Solid‐State Mechanosynthesis of Bipyridine Metal Complexes
Source: Chemistry. 2025 Jun 23;31(38):e202501214. doi: 10.1002/chem.202501214 (PMC12238917; doi:10.1002/chem.202501214)
Supplement: Supplementary file 1 — Supporting Information [file CHEM-31-e202501214-s001.docx]

**SUPPORTING INFORMATION**

**Rapid and Efficient Solid-state Mechanosynthesis of Bipyridine Metal Complexes**

*Talha Munir, ^a^ Eleonora Aneggi, ^a^ Walter Baratta, ^a^ Leonardo Genesin, ^a^ Fabio Trigatti ^a, b^ and Daniele Zuccaccia ^a^ **

*^a^* Dipartimento di Scienze Agroalimentari, Ambientali e Animali, Sezione di Chimica, Università di Udine, Via Cotonificio 108, I-33100 Udine, Italy;

*^b^* Dipartimento di Scienze della Vita, Università di Trieste, Via Weiss 2, 34128 Trieste, Italy

Email: [daniele.zuccaccia@uniud.it](mailto:daniele.zuccaccia@uniud.it);

**Contents**

**Part 1:** General Procedure used for Mechanochemical synthesis

**Part 2:** Details of Trails

**Part 3:** Calculations for E-factor and Effective Mass Yield (EMY).

**Part 4:** NMR spectra’s

**Part 5:** XRD analysis

**Part 1: General procedure for Mechanochemical Synthesis:**

Mechanochemical synthesis of all of the products (1-11) has been carried out with the same approach by means of a vibrational mill (Figure S1). For all, relevant ratios of the metallic precursors (highlighted in the tables from 1-11) are used and milled at required frequencies, depending upon their interaction. The exact milling frequency and time of milling with the resultant output has been highlighted in the table for each product. For most of our synthesis, we used 2ml Eppendorf with 4 balls inside (diameter 3mm/~0.4g) (Figure S2). To avoid oxidation to occur during milling, we closed the Eppendorf in the presence of argon and tightened it with parafilm. However, in some particular cases, we also used 25ml Zirconia jar with one Zr ball (15mm/~11.4g) (Figure S2).


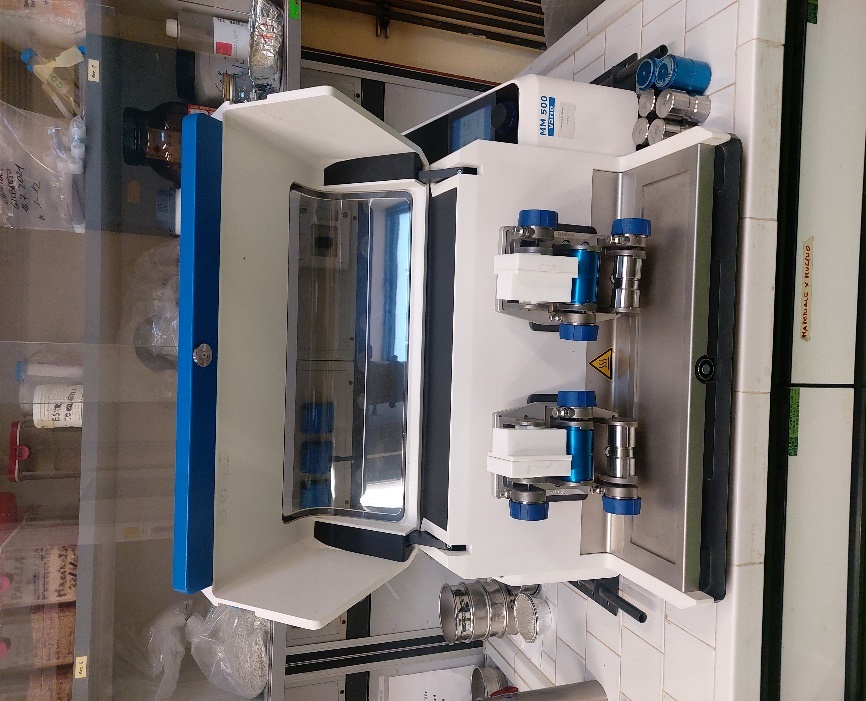


Figure S1: Retsch Ball Mill (MM 500 Vario)


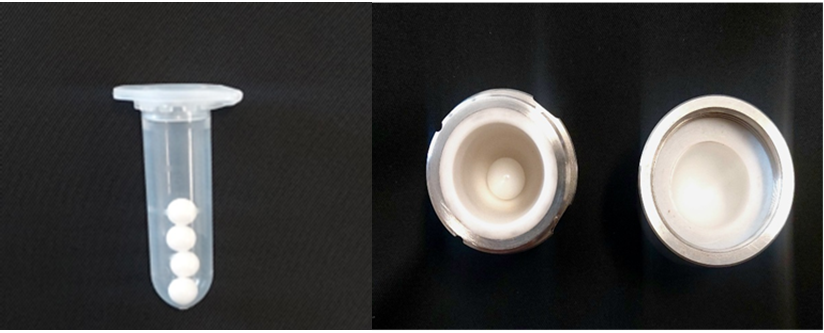


Figure S2: Milling Container

**Part 2: Detail of Trails**

| [Ru(p-Cym)(BiPy)Cl]Cl (1) | | | | | |
| --- | --- | --- | --- | --- | --- |
| Sr | **Time**  **(Min)** | **Frequency**  **(Hz)** | **Ratio** | **Washing** | **Comments** |
| 7839 | 2 | 30 | 1:2 | No | 23% of conversion |
| 7840 | 5 | 30 | 1:2 | No | 33% of conversion |
| 2901 | 10 | 20 | 1:2.1 | No | Completed (0.1% BiPy was washed with pentane) |
| 4903 | 10 | 30 | 1:2 | No | Reaction Completed |
| 3006 | 10 | 20 | 1:2 | No | 82% of conversion |
| 7802* | 30 | 30 | 1:2.05 | No | Reaction Completed |
| 7803* | 50 | 30 | 1:2.05 | No | Reaction Completed (slight excess of BiPy was washed with pentane) |
| 11001** | 2-10 | 30Hz | 1:2 | No | The conversions were observed to be same as that of non-pre milled samples |

*Trails with bulk synthesis (~300mg) of **1** in 25ml Zirconia jar containing 25 balls of zirconia (3mm/~0.4g).

** Eppendorf and balls were milled (without the reactant) for 10 minutes, then reactants were added to observe temperature acceleration of the reaction.

Table S1: Details of the trails for [Ru(p-Cym)(BiPy)Cl]Cl **(1)**

| [Ru(BiPy)(DMSO)_2_Cl_2_] (2) | | | | | |
| --- | --- | --- | --- | --- | --- |
| Sr | **Time** | **Frequency** | **Ratio** | **Washing** | **Comments** |
| 102 | 30 | 30 | 1:1 | No | Reaction Completed |
| 202 | 20 | 20 | 1:1 | Yes | One product+DMSO |
| 203 | 20 | 20 | 1:1 | Vacuum | DMSO present |
| 303 | 120 | 20 | 1:1 | Pentane | Number of products |
| 403 | 60 | 30 | 1:1 | Pentane | Number of products |
| 3105 | 3hours | 30 | 1:3 | No | Number of products |
| 3110 | 3hours | 30 | 1:3 | Pentane | Number of products |

Table S2: Details of trails for [Ru(BiPy)(DMSO)_2_Cl_2_] **(2)**

| [Ru(BiPy)Cl_2_(CO)_2_] (3) | | | | | | |
| --- | --- | --- | --- | --- | --- | --- |
| Sr | **Time**  **(Min)** | **Frequency**  **(Hz)** | **Ratio** | **Washing** | **Comments** | **% Composition** |
| 7810 | 15 | 30 | 1:2 | No | 80% of conversion | All four products were present |
| 7710 | 30 | 30 | 1:2 | No | completed | All products were present |
| 204 | 20 | 20 | 1:2 | Pentane |  | 3d in highest concentration |
| Interconversion of Isomers | | | | | | |
| 708 | 10 | 10 | 1:2 | Pentane | 3 products (**3c** in high conc.) | 78% (**3c**)-9.5%(**3b**)-8.6%(**3a**) |
| 801 | 05 | 10 | 1:2 | Pentane | 2 products | 77% (**3c**), 22% (**3b**) |
| 804 | 1:2 (above tube after 3 days in tube)  Pentane | | | | No interconversion due to solution | 77% (**3c**), 22% (**3b**) |
| 1501 | 30 | 10 | 1:2.1 | Pentane | 3 products (**3c** in high conc.) | 70%(**3c**)-17.9%(**3b**)- 11.75%(**3a**) |
| 1502 | 60 | 10 | 1:2.1 | Pentane |  | 59.3%(**3c**)-22.8%(**3b**)-17.8%(**3a**) |
| 1503 | 90 | 10 | 1:2.1 | Pentane | Ionic product (**3d**) in high conc. | 55%(**3d**), 26%(**3c**), 12.5%(**3b**), 6%(**3a**) |
| 1504 | 120 | 10 | 1:2.1 | Pentane | Ionic product (**3d**) in high conc. | 61%(**3d**), 22%(**3c**), 10%(**3b**), 6%(**3a**) |
| 1505 | 150 | 10 | 1:2.1 | Pentane | Ionic product (**3d**) in high conc. | 53%(**3d**), 28.5%(**3c**), 11.5%(**3b**), 6.5%(**3a**) |
| 1506 | 180 | 10 | 1:2.1 | Pentane | Ionic product (**3d**) in high conc. | 61%(**3d**), 21.3%(**3c**), 10.1%(**3b**), 7.3%(**3a**) |
| 3028 |  | No | 1:2.1 |  |  | 93% of free BiPy |

Table S3: Details of Trials for [Ru(BiPy)Cl_2_(CO)_2_] **(3)**

| [RuCl_2_(PPh_3_)_2_(BiPy)] (4a, 4b)/ 20Hz | | | | | | | | | |
| --- | --- | --- | --- | --- | --- | --- | --- | --- | --- |
| Sr | Time  (min) | Frequency  (Hz) | | | Balls | Ratio | | Washing | Comments |
| 1001 | 05 | 20 | | | 4 | 1:1 | |  |  |
|  |  |  |  |  |  |  |  | Pentane | No product |
| 1003 | 10 | 20 | | | 4 | 1:1 | |  |  |
|  |  |  |  |  |  |  |  | Pentane | No product |
| 1005 | 20 | 20 | | | 4 | 1:1 | |  |  |
|  |  |  |  |  |  |  |  | Pentane | No product |
| 1204 | 30 | 20 | | | 4 | 1:1 | |  | No Product |
|  |  |  |  |  |  |  |  | Pentane |  |
| 1008 | 60 | 20 | | | 4 | 1:1 | |  |  |
|  |  |  |  |  |  |  |  | Pentane | No Product |
| 1208 | 90 | 20 | | | 4 | 1:1 | |  | Conversion of 75% (**4a**) |
|  |  |  |  |  |  |  |  | Pentane | **(4a)** |
| 1009 | 120 | 20 | | | 4 | 1:1 | |  |  |
|  |  |  |  |  |  |  |  | Pentane | Conversion from **4a** to **4b** starts (95% and 5% respectively) |
| 1011 | 180 | 20 | | | 4 | 1:1 | |  |  |
|  |  |  |  |  |  |  |  | Pentane | Increasing Conversion from **4a** to **4b** (40% and 60% respectively) |
| 2401 | 20 | 20 | | | 25 mL Jar | 1:1 | | Pen | High conversion and energy (61% of **4a** and 39% of **4b**) |
| 1910 | 180 | 20 | | | 4 | 1:1 | |  | High conversion but start decomposition |
| (1915) | 180 | 20 | | | 4 | 1:1 | | Pentane | 98% **4a**, 2%PPh_3_O+ Decomposition |
| 1916 | 240 | 20 | | | 4 |  | | 1911 | High conversion Decomposition Increases  (10% **4b**, 45% **4a** and 44% Ru-Precursor) |
|  |  |  |  |  |  |  |  | Pentane | 97% of **4a** and 3% of **4b** |
| [RuCl_2_(PPh_3_)_2_(BiPy)] (4a, 4b)/ 30Hz | | | | | | | | | |
| Sr | **Time** | | **Freq** | **Balls** | | | **Ratio** | **Washing** | **Comments** |
| 901 | 05 | | 30 | 4 | | | 1:1.1 | 3001 | 19% Ru-Precursor), 63% PPh_3_O, 17% of **4a** |
|  |  |  |  |  |  |  |  | Pentane | **4a** and PPh_3_O |
| 903 | 10 | | 30 | 4 | | | 1:1 |  |  |
|  |  |  |  |  |  |  |  | Pentane | **4b** starts to appear  10% **4a**, 4% **4b**, 85% PPh_3_O |
| 905 | 20 | | 30 | 4 | | | 1:1 |  |  |
|  |  |  |  |  |  |  |  | Pentane | **4b** increases  9% 4a, 6%4b, 85% PPh_3_O |
| 2001 | 30 | | 30 | 4 | | | 1:1 |  | Both present (44%free, 42%4a, 8%4b and 3% PPh_3_O |
| 2013 | 30 | | 30 | 4 | | | 1:1 | Pentane | Almost pure mixture of **4a** and **4b**  87% **4a**, 8.5% **4b**, and 4% PPh_3_O |
| 2109 | 30+30 | | 30 | 4 | | | 1:1 |  | High interconversion of 4a to 4b along with decomposition  34% Ru-Precursor, 26% **4a**, 11% PPh_3_O, 28% **4b** |
| 2123 | 30+30 | | 30 | 4 | | | 1:1 | Pentane | 31% **4a**, 69% **4b** |
| 909 | 120 | | 30 | 4 | | | 1:1 |  |  |
|  |  |  |  |  |  |  |  | Pen | 76% 4a, 15% PPh_3_O, 8%4b |
| 2404 | 20 | | 30 | 25 mL Jar | | | 1:1 | Pen | High energy with jar  44% **4a**, 17% PPh_3_O, 37% **4b** |

Table S4: Details of Trials for RuCl_2_(PPh_3_)_2_(BiPy)] (4a, 4b)

| Ru[COD][BiPy]Cl_2_ (5) | | | | | |
| --- | --- | --- | --- | --- | --- |
| Sr | Time  (min) | Frequency  (Hz) | Ratio | Washing | Comment |
| 3206 | 05 | 30 | 1:1 | No | 55% of conversion |
| 7816 | 10 | 30 | 1:1 | No | 71% of conversion |
| 7824 | 15 | 30 | 1:1 | No | 73% of conversion |
| 3630 | 20 | 30 | 1:1 | No | 76% of conversion |
| 7818 | 30 | 30 | 1:1 | No | Reaction Completed |
| 3304 | 45 | 30 | 1:1 | No | Reaction Completed |
| 3305 | 60 | 30 | 1:1 | No | Reaction Completed |
| 3104 | 90 | 30 | 1:1 | No | Reaction Completed |
| 2407 | 30 | 30 | 1:2 | No | 62% of free BiPy |
| 2408 | 30 | 30 | 1:3 | No | 77% of free BiPy |
| 11002 | 30 | 30 | 1:1 | No | The conversions were observed to be same as that of non-pre milled samples |

Table S5: Details of Trials for Ru[COD][BiPy]Cl_2_ **(5)**

* Eppendorf and balls were milled (without the reaction mixture) for 30min, then reagents were added to observe temperature acceleration.

| Pd(BiPy)Cl_2_ (6) | | | | | |
| --- | --- | --- | --- | --- | --- |
| Sr | **Time**  **(Min)** | **Frequency**  **(Hz)** | **Ratio** | **Washing** | **Comments** |
| 2515 | 20 | 20 | 1:1.1 | No | 85% of conversion |
| 2516 | 60 | 30 | 1:1.1 | No | 87% of conversion |
| 2703 | 05 | 15 | 1:1 | No | 83% of conversion |
| 3015 | 120 | 20 | 1:1 | No | Reaction Completed |

Table S6: Details of trials for Pd(BiPy)Cl_2_ **(6)**

| Pd(BiPy)I_2_ (7) | | | | | |
| --- | --- | --- | --- | --- | --- |
| Sr | **Time**  **(Min)** | **Frequency**  **(Hz)** | **Ratio** | **Washing** | **Comments** |
| 7822 | 30 | 30 | 1:1 | No | 60% of conversion |
| 7823 | 60 | 30 | 1:1 | No | Reaction Completed |
| 7808 | 120 | 15 | 1:1 | No | Reaction Completed |

Table S7: Details of trials for Pd(BiPy)I_2_ **(7)**

| Pt(BiPy)Cl_2_ (8) | | | | | |
| --- | --- | --- | --- | --- | --- |
| Sr | Time  (Min) | Frequency  (Hz) | Ratio | Washing | Comments |
| 2701 | 05 | 15 | 1:1.1 | No | 15% of conversion |
| 2702 | 05 | 15 | 1:1.1 | Pentane | BiPy removed |
| 2814 | 10 | 15 | 1:1.1 | No | 53% of conversion |
| 2910 | 15 | 15 | 1:1.1 | No | 61% of **8**, 38% of other product |
| 2915 | 15 | 15 | 1:1.1 | Pentane | 61% of **8**, 38% of other product gets reduced to 17%, |
| 2423 | 30 | 30 | 1:1.1 | Pentane | 30% of other product |
| 3016 | 120 | 20 | 1:1 | No | 77% of **8**, and 23% of other product |
| 3018 | 120 | 20 | 1:1 | Pentane | 77% of **8**, and 23% of other product |

Table S8: Details of Trials for Pt(BiPy)Cl_2_ **(8)**

| Fe(BiPy)_3_(BF_4_)_2_ (9) | | | | | |
| --- | --- | --- | --- | --- | --- |
| Sr | **Time** | **Frequency** | **Ratio** | **Washing** | **Comments** |
| 2707 | 30 | 30 | 1:3.1 | No | Reaction Completed |
| 2708 | 30 | 30 | 1:3.1 | Pentane | Reaction Completed |
| 2906 | 15 | 15 | 1:3.1 |  | Reaction Completed |
| 2916 | 15 | 15 | 1:3.1 | Pentane | Reaction Completed |
| 3005 | 15 | 15 | 2:6.2 |  | 20% of free BiPy |
| 2918 | Just Mixing | | 1:3 |  | 35% of conversion |

Table S9: Details of Trials for Fe(BiPy)_3_(BF_4_)_2_ **(9)**

| *Co(BiPy)_3_Cl_2_(10)* | | | | | |
| --- | --- | --- | --- | --- | --- |
| Sr | Time | Frequency | Ratio | Washing | Comments |
| 1 | 30 | 30 | 1:3.01 | No | Reaction Completed |
| 2* | 60 | 30 | 1:3 | No | Reaction Completed |

Table S10: Details of Trials for Co(BiPy)_3_Cl_2_ **(10)**

*The reaction was carried out in 25ml Zr jar with a Zr Ball (15mm/~11.4g)

| [Ir(Cp*)(BiPy)Cl]Cl (11) | | | | | |
| --- | --- | --- | --- | --- | --- |
| Sr | Time | Frequency | Ratio | Washing | Comments |
| 2406 | 30 | 30 | 1:2.1 | No | Reaction Completed |
| 2907 | 20 | 20 | 1:2.1 |  | 80% of conversion |
| 3007 | 20 | 20 | 1:2.1  Double amount |  | 75% of conversion |

Table S11: Details of Trials for [Ir(Cp*)(BiPy)Cl]Cl **(11)**

**Part 3: Calculations for E-factor and Effective Mass Yield (EMY).**

E-factor and (EMY) are calculated according to the following expressions.

$$E-Factor=\frac{Mass of Waste}{Mass of Product}$$

$$EMY= \frac{Mass of Product}{Total Mass of Material Used} x 100$$

| [Ru(p-Cym)(BiPy)Cl]Cl (1) | |
| --- | --- |
| Solution Synthesis | |
| Reactant | **Amount (g)** |
| Ru-precursor | 0.15 |
| BiPy | 0.076 |
| KPF_6_ | 0.09 |
| Total: | 0.316 |
| Percentage yield obtained in solution | 71 |
| Amount of product obtained | 0.224 |
| Waste | 0.092 |
| EMY | **71** |
| E-factor | **0.408** |
| Mechanochemical Synthesis | |
| Reactant | **Amount (g)** |
| Ru-Precursor | 0.015 |
| BiPy | 0.008 |
| Washing | No |
| Total: | 0.023 |
| Percentage Yield Obtained | 100 |
| Amount of Product obtained | 0.023 |
| Waste | 0 |
| EMY | **~100** |
| E-factor | **~0** |

Table S12: Green Metrics Calculation for [Ru(p-Cym)(BiPy)Cl] **(1)**

| [Ru(BiPy)(DMSO)_2_Cl_2_] (2) | |
| --- | --- |
| Mechanochemical Synthesis | |
| Reactant | **Amount (g)** |
| Ru-Precursor | 0.015 |
| BiPy | 0.005 |
| Total: | 0.02 |
| Mass of Side Product (2 DMSO) | 0.005 |
| Percentage Yield Obtained | 100 |
| Amount of Product obtained | 0.015 |
| Waste | 0.005 |
| EMY | **75.6** |
| E-factor | **0.323** |

Table S13: Green Metrics Calculations for [Ru(BiPy)(DMSO)_2_Cl_2_] **(2)**

| [Ru(BiPy)Cl_2_(CO)_2_] (3) | |
| --- | --- |
| Solution Synthesis | |
| Reactant | **Amount (g)** |
| Ru-precursor | 0.741 |
| BiPy | 0.609 |
| Methanol (20ml) | 15.8 |
| Total: | 17.15 |
| Percentage yield obtained in solution | 72% |
| Amount of product obtained | 0.975 |
| Waste | 16.175 |
| EMY | **5.6851** |
| E-factor | **16.59** |
| Mechanochemical Synthesis | |
| Reactant | **Amount (g)** |
| Ru-Precursor | 0.015 |
| BiPy | 0.0092 |
| Total: | 0.0242 |
| Percentage Yield Obtained | 100 |
| Amount of Product obtained | 0.0242 |
| Waste | 0 |
| EMY | **100** |
| E-factor | **~0** |

Table S14: Green Metrics Calculation for [Ru(BiPy)Cl_2_(CO)_2_] **(3)**

| [RuCl_2_(PPh_3_)_2_(BiPy)] (4a, 4b) | |
| --- | --- |
| Solution Synthesis | |
| Reactant | **Amount (g)** |
| Ru-precursor | 0.05 |
| BiPy | 0.01 |
| Acetone (20ml) | 15.7 |
| Washing (10ml of Diethyl Ether) | 7.06 |
| Total: | 22.82 |
| Percentage yield obtained in solution | 90 |
| Amount of product obtained | 0.04 |
| Waste | 22.72 |
| EMY | **0.1753** |
| E-factor | **569.5** |
| Mechanochemical Synthesis | |
| Reactant | **Amount (g)** |
| Ru-Precursor | 0.015 |
| BiPy | 0.0024 |
| Washing (6 ml pentane) | 3.75 |
| Total: | 3.7674 |
| Percentage Yield Obtained | 53 |
| Percentage of PPh_3_O | 4 |
| Amount of Product obtained | 0.00705 |
| Amount of PPh_3_O Obtained | 0.0017 |
| Waste | 3.76035 |
| EMY | **0.18** |
| E-factor | **534.38** |

Table S15: Green Metrics Calculation for [RuCl_2_(PPh_3_)_2_(BiPy)] **(4a, 4b)**

| Ru[COD][BiPy]Cl_2_ (5) | |
| --- | --- |
| Mechanochemical Synthesis | |
| Reactant | **Amount (g)** |
| Ru-Precursor | 0.015 |
| BiPy | 0.008 |
| Washing | No |
| Total: | 0.023 |
| Percentage Yield Obtained | 100 |
| Amount of Product obtained | 0.023 |
| Waste | 0 |
| EMY | **~100** |
| E-factor | **~0** |

Table S16: Green Metrics Calculations for Ru[COD][BiPy]Cl_2_ **(5)**

| *Pd(BiPy)Cl_2_ (6)* | |
| --- | --- |
| Solution Synthesis | |
| Reactant | **Amount (g)** |
| Ru-precursor | 0.266 |
| BiPy | 0.234 |
| Methanol (10ml) | 7.9 |
| Total: | 8.4 |
| Percentage yield obtained in solution | 88% |
| Amount of product obtained | 0.44 |
| Waste | 7.96 |
| EMY | **5.23** |
| E-factor | **18.091** |
| Mechanochemical Synthesis | |
| Reactant | **Amount (g)** |
| Ru-Precursor | 0.015 |
| BiPy | 0.013 |
| Washing | No |
| Total: | 0.028 |
| Percentage Yield Obtained | 100 |
| Amount of Product obtained | 0.028 |
| Waste | 0 |
| EMY | **~100** |
| E-factor | **~0** |

Table S17: Green Metrics Calculation for Pd(BiPy)Cl_2_ **(6)**

| *Pd(BiPy)I_2_ (7)* | |
| --- | --- |
| Mechanochemical Synthesis | |
| Reactant | **Amount (g)** |
| Pd-Precursor | 0.015 |
| BiPy | 0.065 |
| Washing | No |
| Total: | 0.0215 |
| Percentage Yield Obtained | 100 |
| Amount of Product obtained | 0.0215 |
| Waste | 0 |
| EMY | **~100** |
| E-factor | **~0** |

Table S18: Green Metrics Calculations for Pd(BiPy)I_2_ (7)

| *Pt(BiPy)Cl_2_ (8)* | |
| --- | --- |
| Solution Synthesis | |
| Reactant | **Amount (g)** |
| Pt-Precursor | 1.44 |
| BiPy | 0.541 |
| HCl | 5.06 |
| water | 10 |
| Total: | 17.04 |
| Percentage Yeild Obtained In solution | 92 |
| Amount of Product obtained | 1.84 |
| Waste | 15.2 |
| EMY | **10.8** |
| E-factor | **8.261** |
| Mechanochemical Synthesis | |
| Reactant | **Amount (g)** |
| Pt-Precursor | 0.015 |
| BiPy | 0.009 |
| Percentage Yield Obtained | 100 |
| Amount of Product obtained | 0.024 |
| EMY | **~100** |
| E-factor | **~0** |

Table S19: Green Metrics Calculations for Pt(BiPy)Cl_2_ (8)

| *Fe(BiPY)_3_(BF_4_)_2_ (9)* | |
| --- | --- |
| Mechanochemical Synthesis | |
| Reactant | **Amount (g)** |
| Fe-Precursor | 0.015 |
| BiPy | 0.02 |
| Washing | No |
| Total: | 0.035 |
| Percentage Yield Obtained | 100 |
| Amount of Product obtained | 0.035 |
| Waste | 0 |
| EMY | **~100** |
| E-factor | **~0** |

Table S20: Green Metrics Calculations for Fe(BiPY)_3_(BF_4_)_2_ (9)

| *Co(BiPy)_3_Cl_2_(10)* | |
| --- | --- |
| Mechanochemical Synthesis | |
| Reactant | **Amount (g)** |
| Co-Precursor | 0.015 |
| BiPy | 0.0305 |
| Washing | No |
| Total: | 0.0455 |
| Percentage Yield Obtained | 100 |
| Amount of Product obtained | 0.0455 |
| Waste | 0 |
| EMY | **~100** |
| E-factor | **~0** |

Table S21: Green Metrics Calculations for Co(BiPy)_3_(Cl)_2_ **(10)**

| [Ir(Cp*)(BiPy)Cl]Cl (11) | |
| --- | --- |
| Mechanochemical Synthesis | |
| Reactant | **Amount (g)** |
| Ir-Precursor | 0.015 |
| BiPy | 0.004 |
| washing | No |
| Total: | 0.019 |
| Percentage Yield Obtained | 100 |
| Amount of Product obtained | 0.019 |
| Waste | 0 |
| EMY | **~100** |
| E-factor | **~0** |
| Solution Synthesis | |
| Reactant | **Amount (g)** |
| Ir-Precursor | 1 |
| BiPy | 0.49 |
| DMF (110ml) | 103.8 |
| DEE (150ml) | 107 |
| Total: | 212.3 |
| Percentage Yeild Obtained In solution | 93 |
| Amount of Product obtained | 1.3 |
| Waste | 211 |
| EMY | **~0.612** |
| E-factor | **~162.3** |

Table S22: Green Metrics Calculations for [Ir(Cp*)(BiPy)Cl]Cl (**11)**

**Part 4: NMR spectra’s**


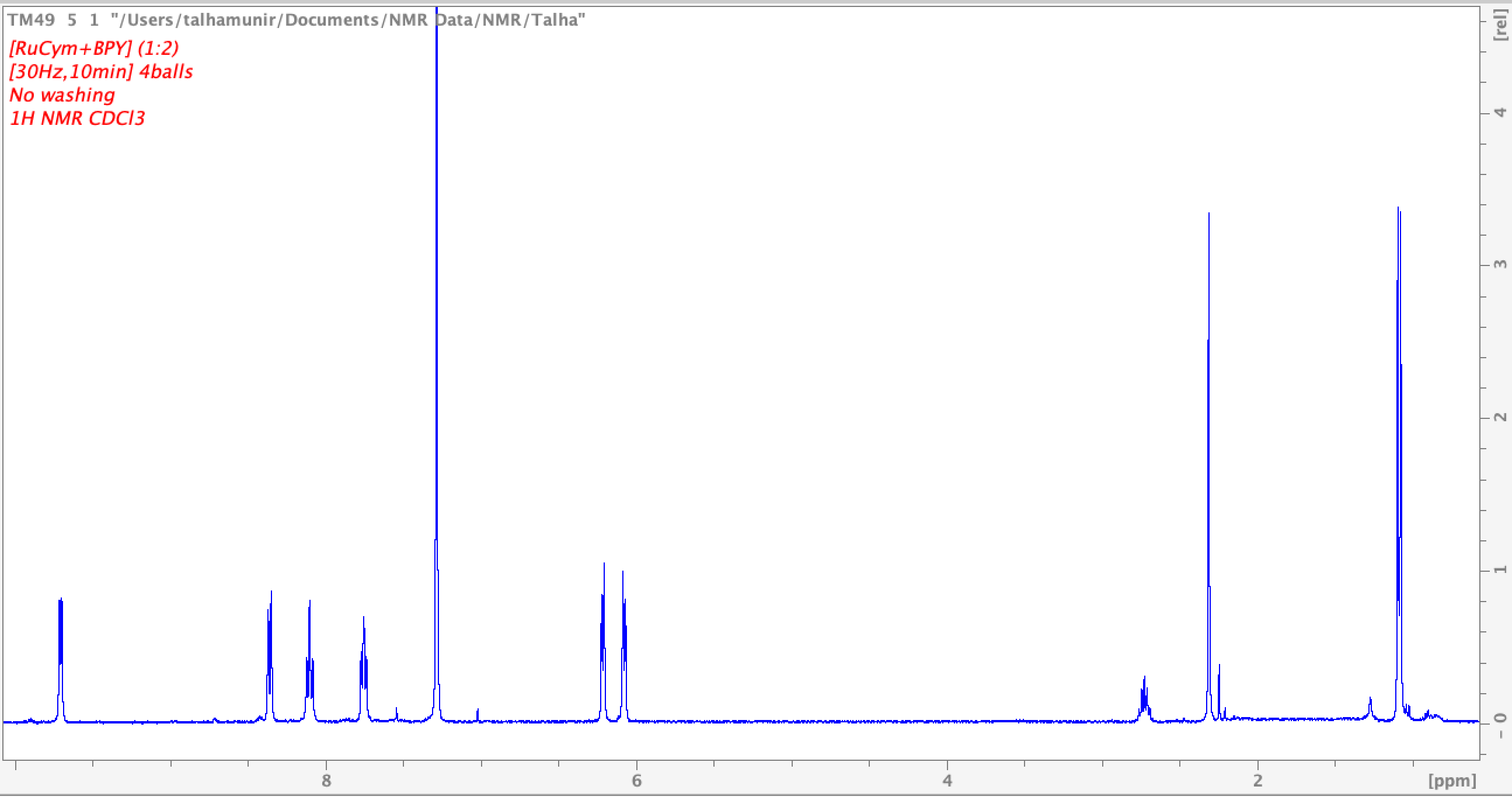


Figure S3: ^1^H-NMR Spectra of [Ru(p-Cym)(BiPy)Cl]Cl **(1)**


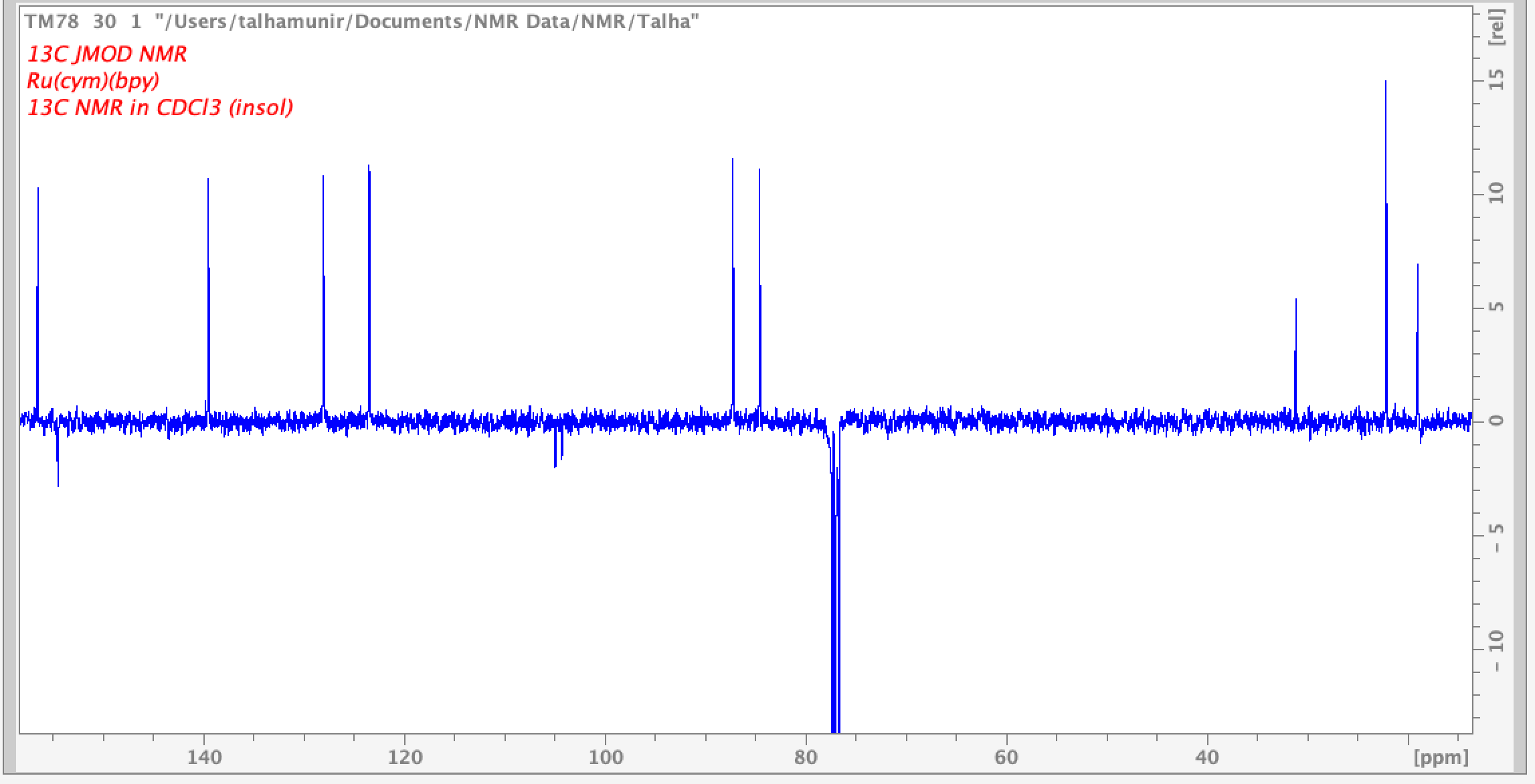


Figure S4: ^13^C-NMR Spectra of [Ru(p-Cym)(BiPy)Cl]Cl **(1)**

Figure S5: ^1^H NMR spectra at different milling time and conversion for complex **(1)**


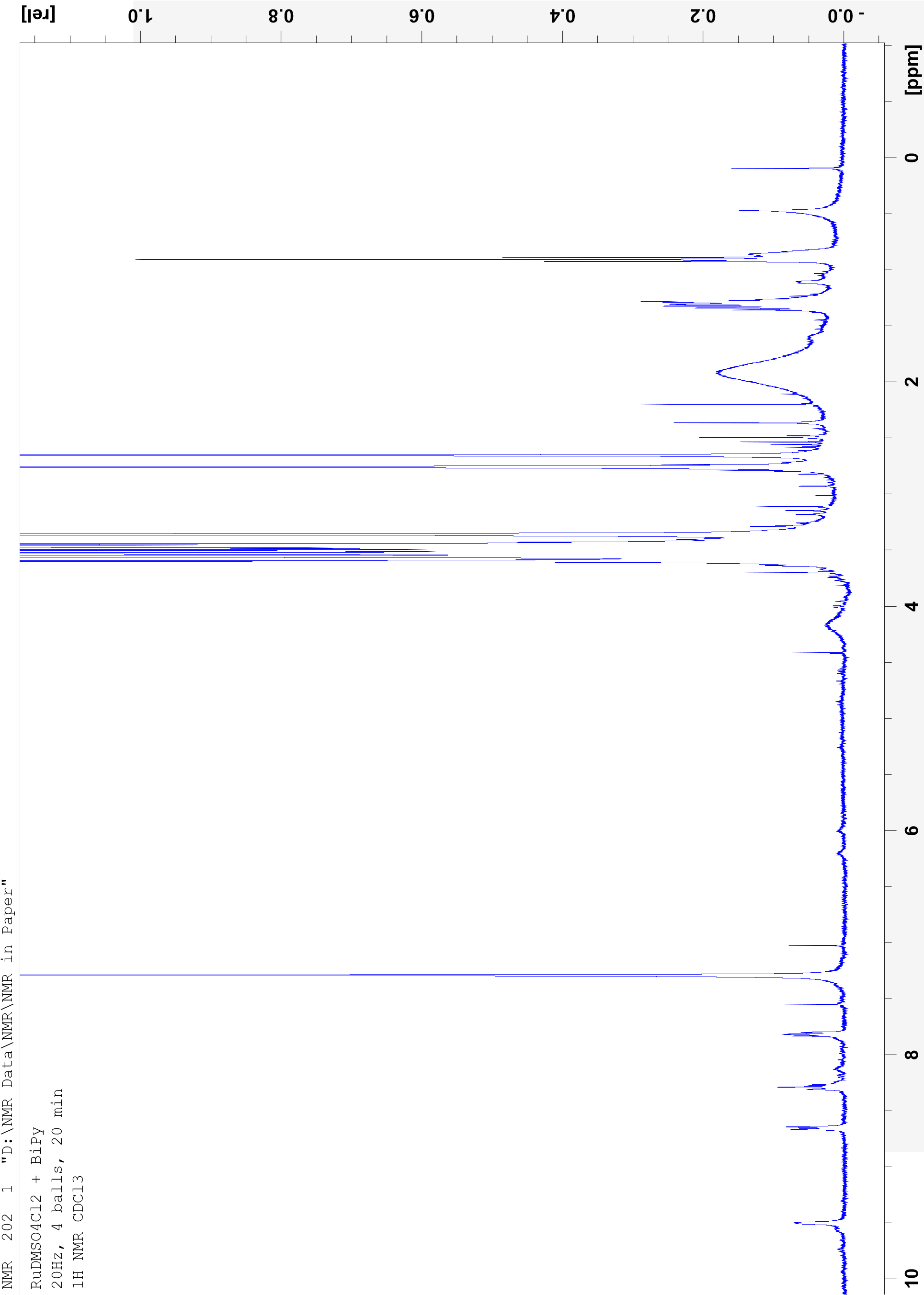


Figure S6: ^1^H-NMR Spectra of [Ru(BiPy)(DMSO)_2_Cl_2_] **(2)**


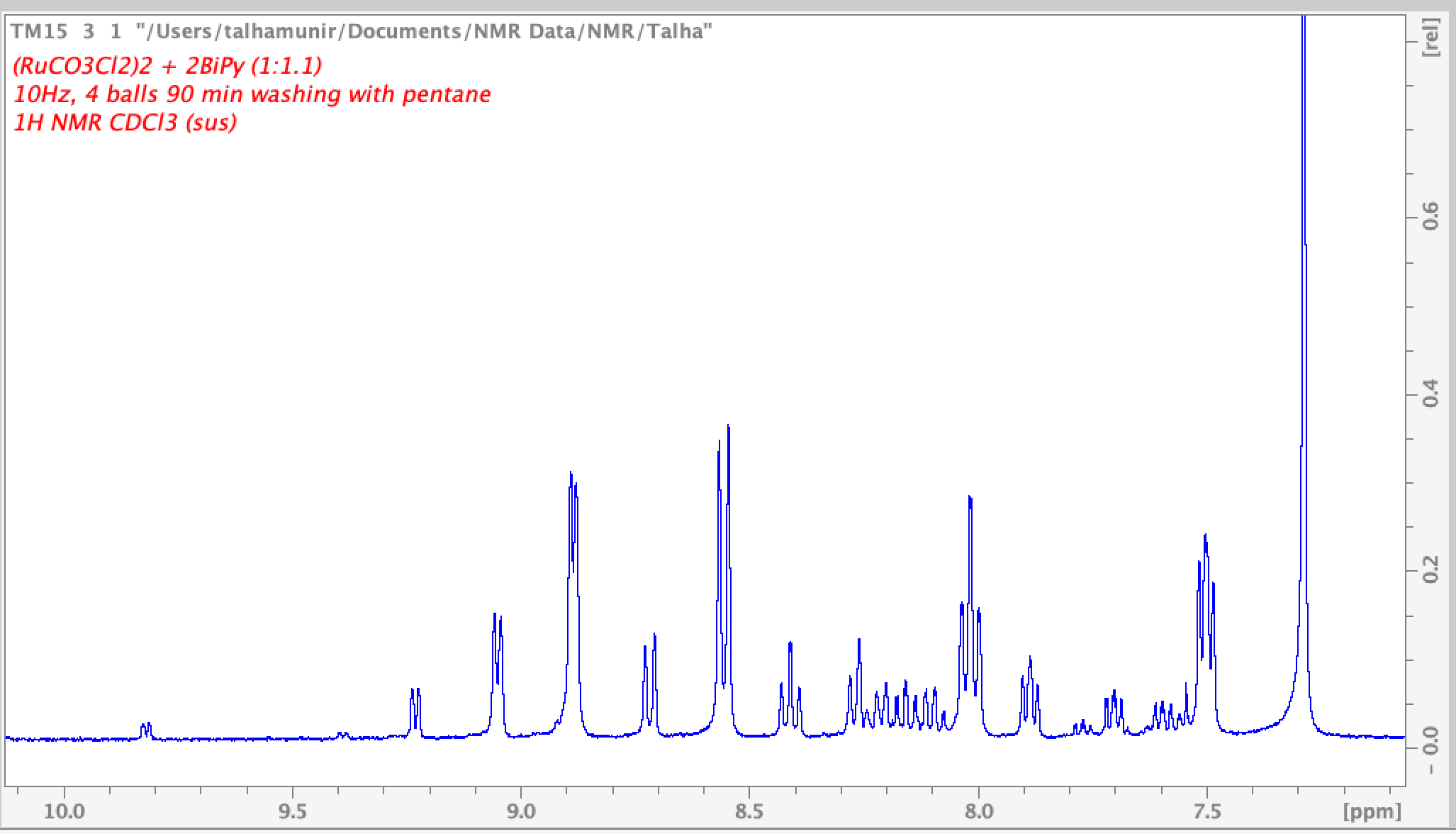


Figure S7: Portion of ^1^H-NMR Spectra of [Ru(BiPy)Cl_2_(CO)_2_] **(3a,3b,3c,3d)**


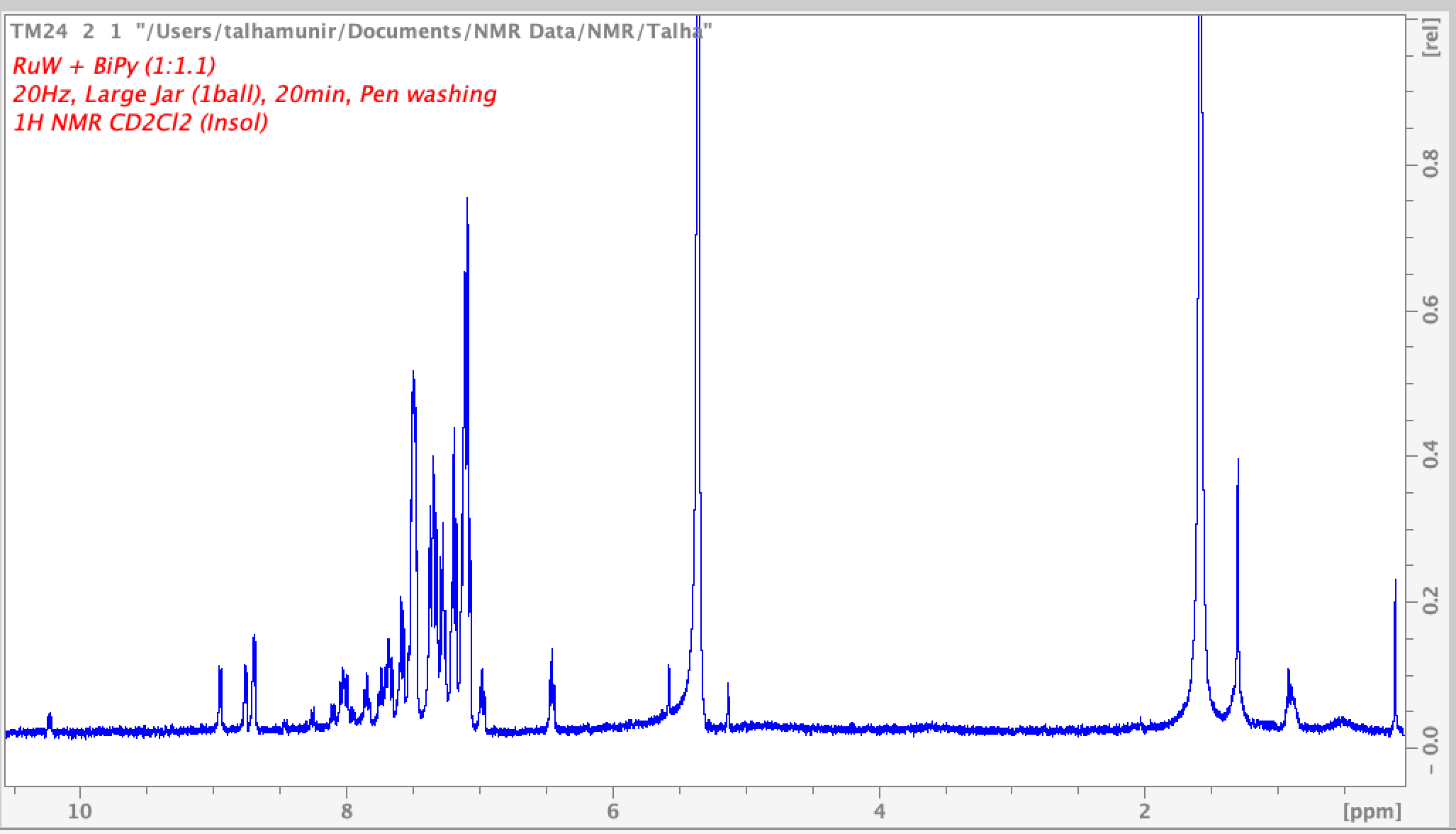


Figure S8: ^1^H-NMR Spectra of [RuCl_2_(PPh3)_2_(BiPy)] **(4a, 4b)**


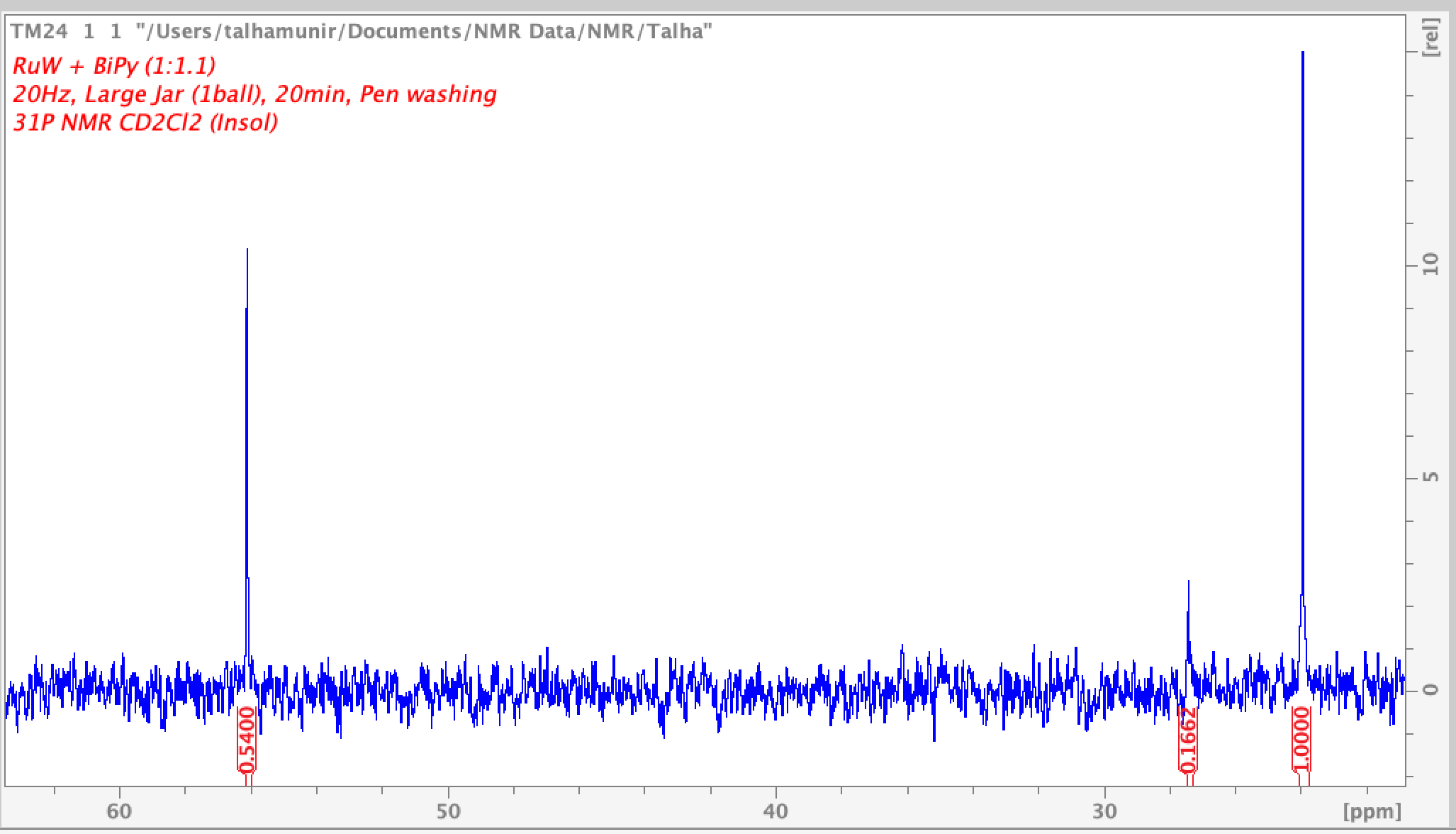


Figure S9:^31^P-NMR Spectra of [RuCl_2_(PPh_3_)_2_(BiPy)] **(4a, 4b)**


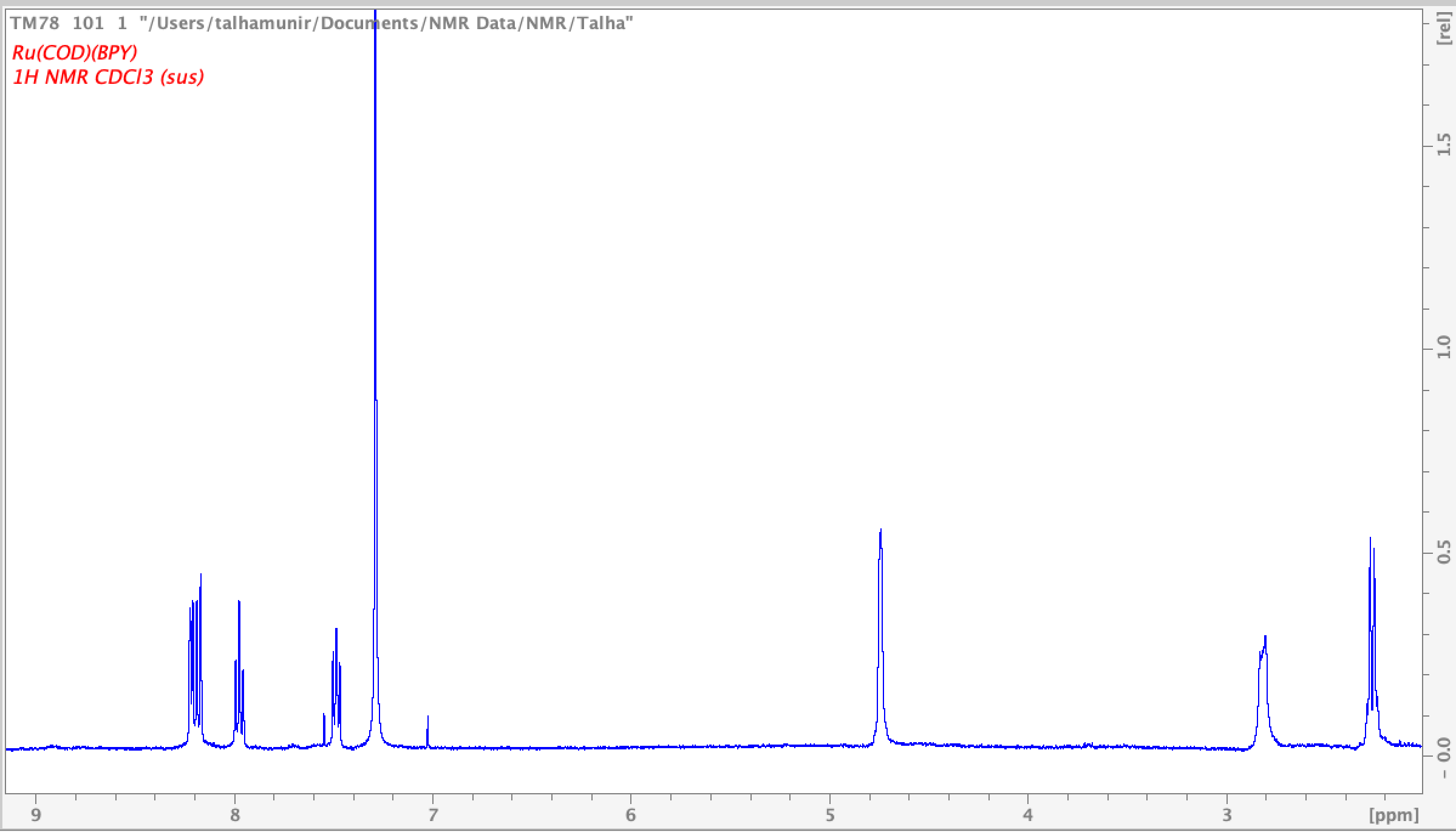


Figure S10: ^1^H-NMR Spectra of Ru[COD][BiPy]Cl_2_ **(5)**


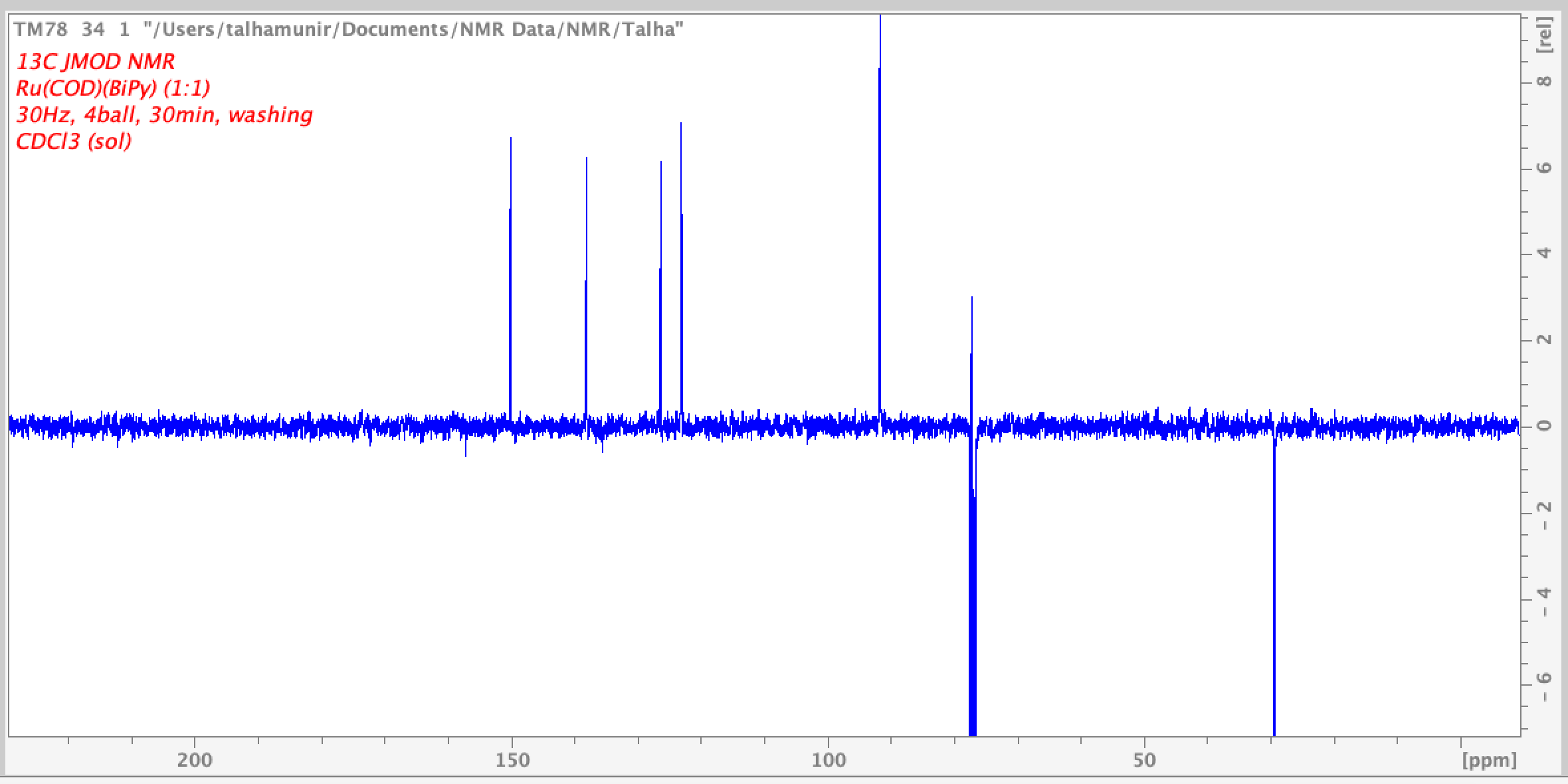


Figure S11: ^13^C-NMR Spectra of Ru[COD][BiPy]Cl_2_ **(5)**


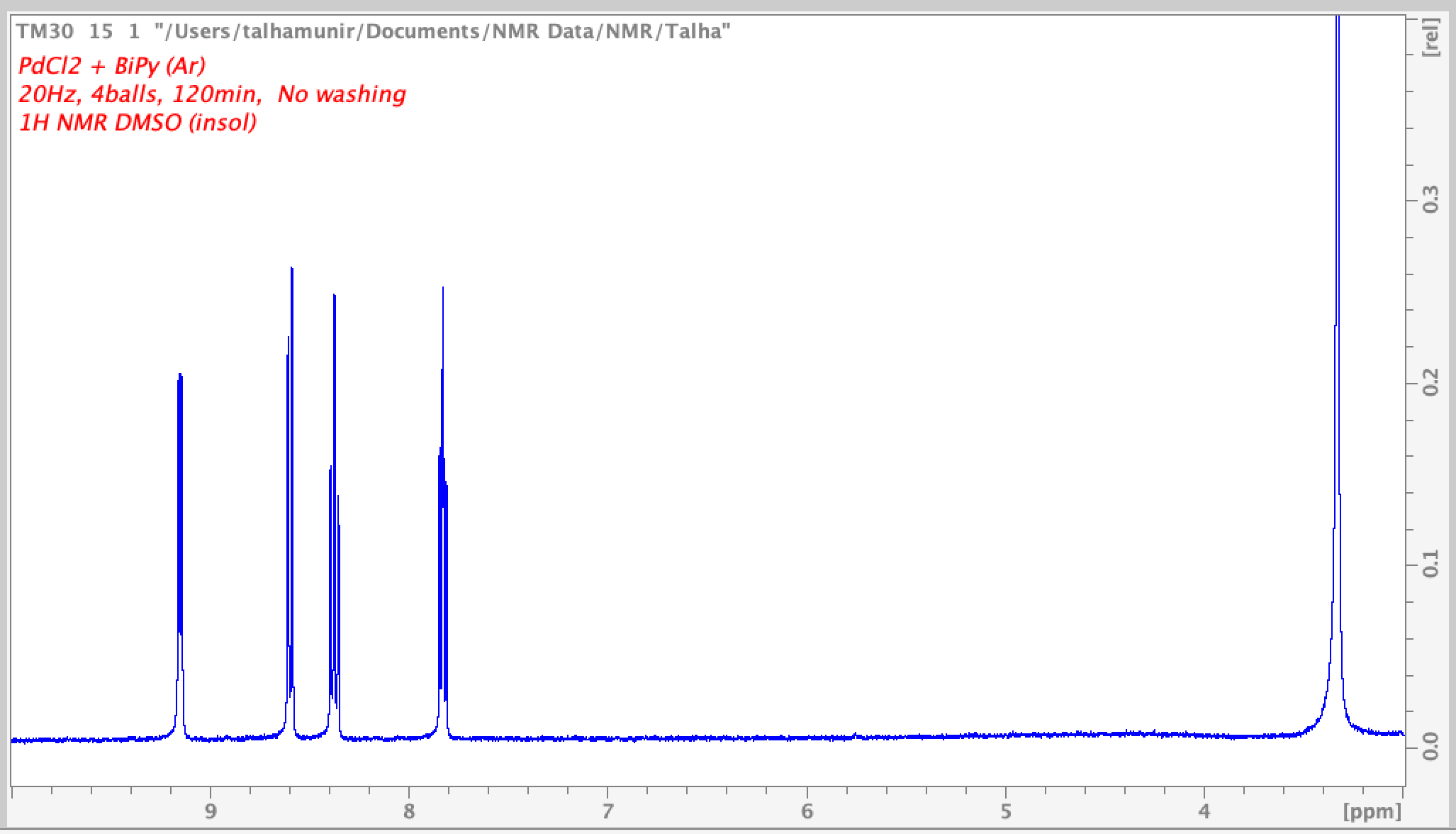


Figure S12: ^1^H-NMR Spectra of Pd(BiPy)Cl_2_ **(6)**


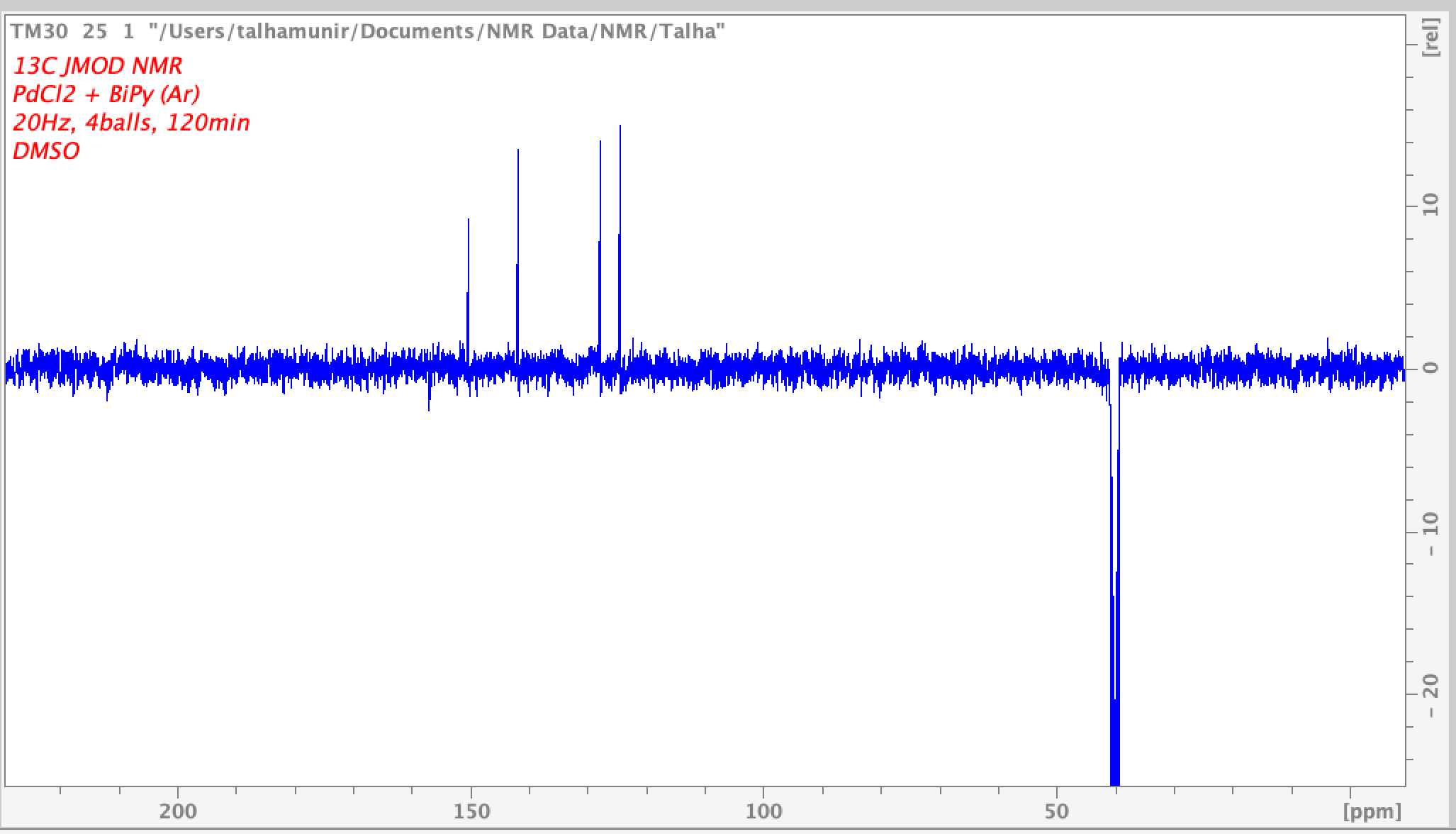


Figure S13: ^13^C-NMR Spectra of Pd(BiPy)Cl_2_ **(6)**


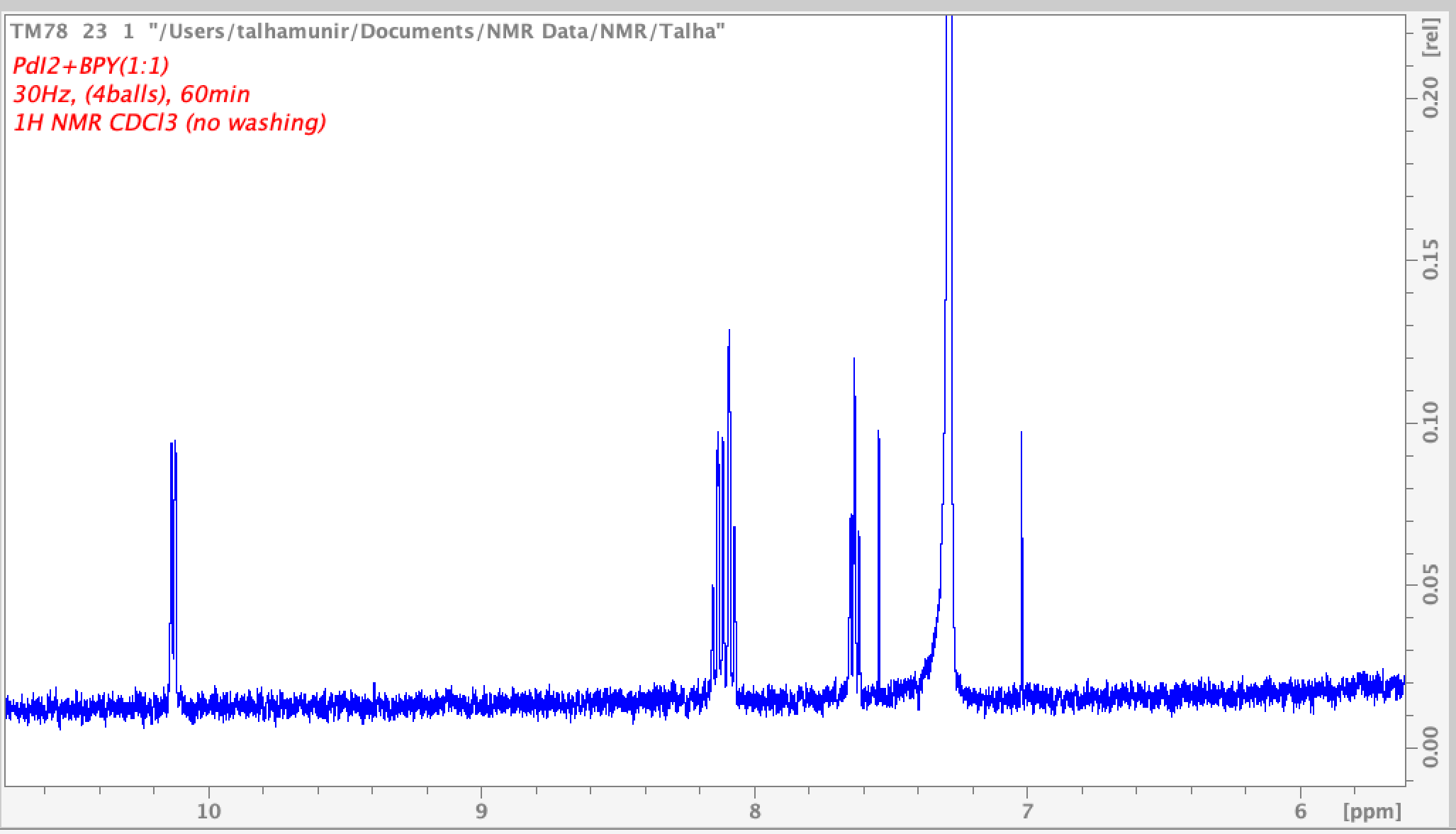


Figure S14: 1H-NMR Spectra of Pd(BiPy)I_2_ **(7)**


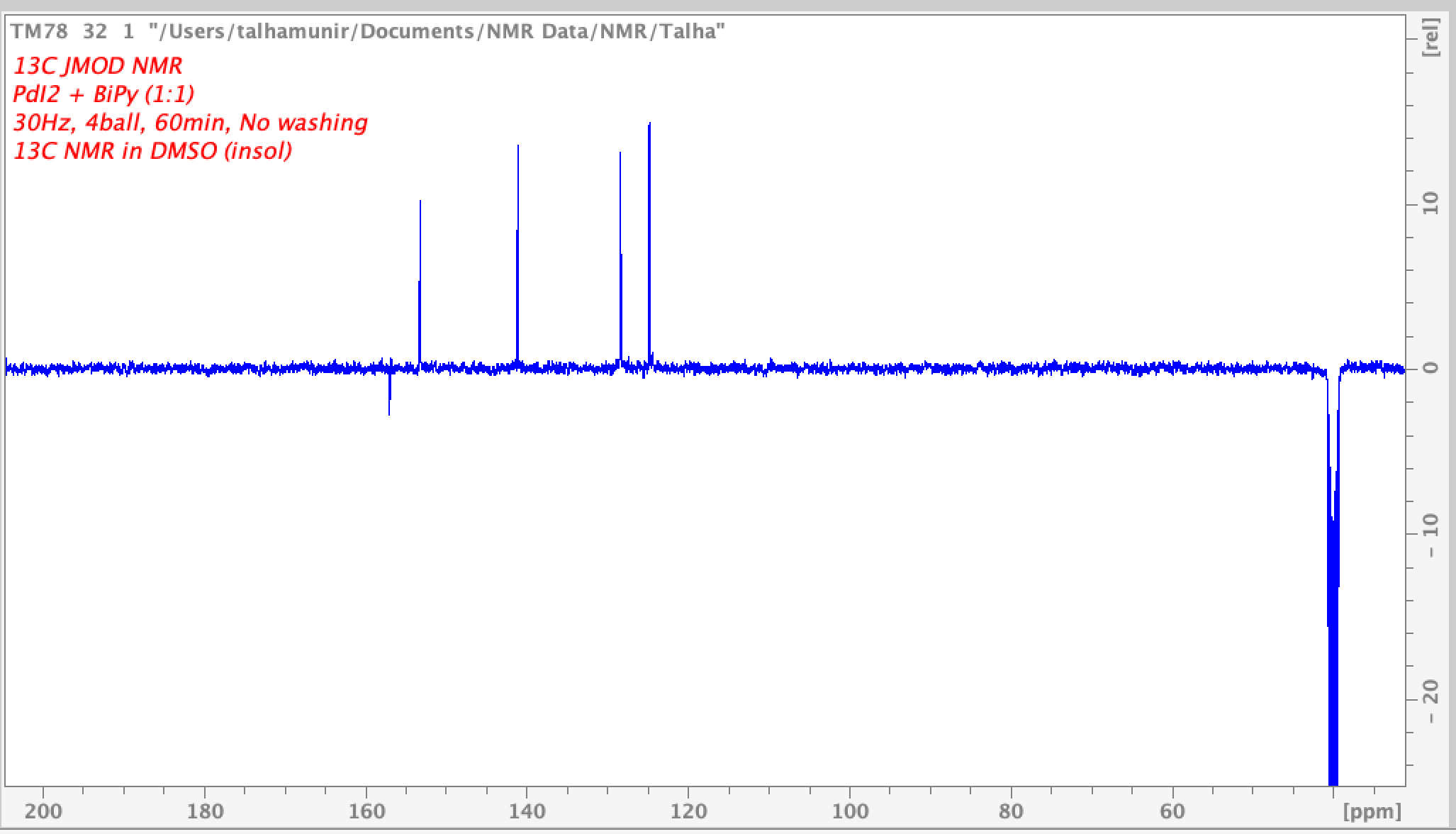


Figure S15: 13C-NMR Spectra of Pd(BiPy)I_2_ **(7)**


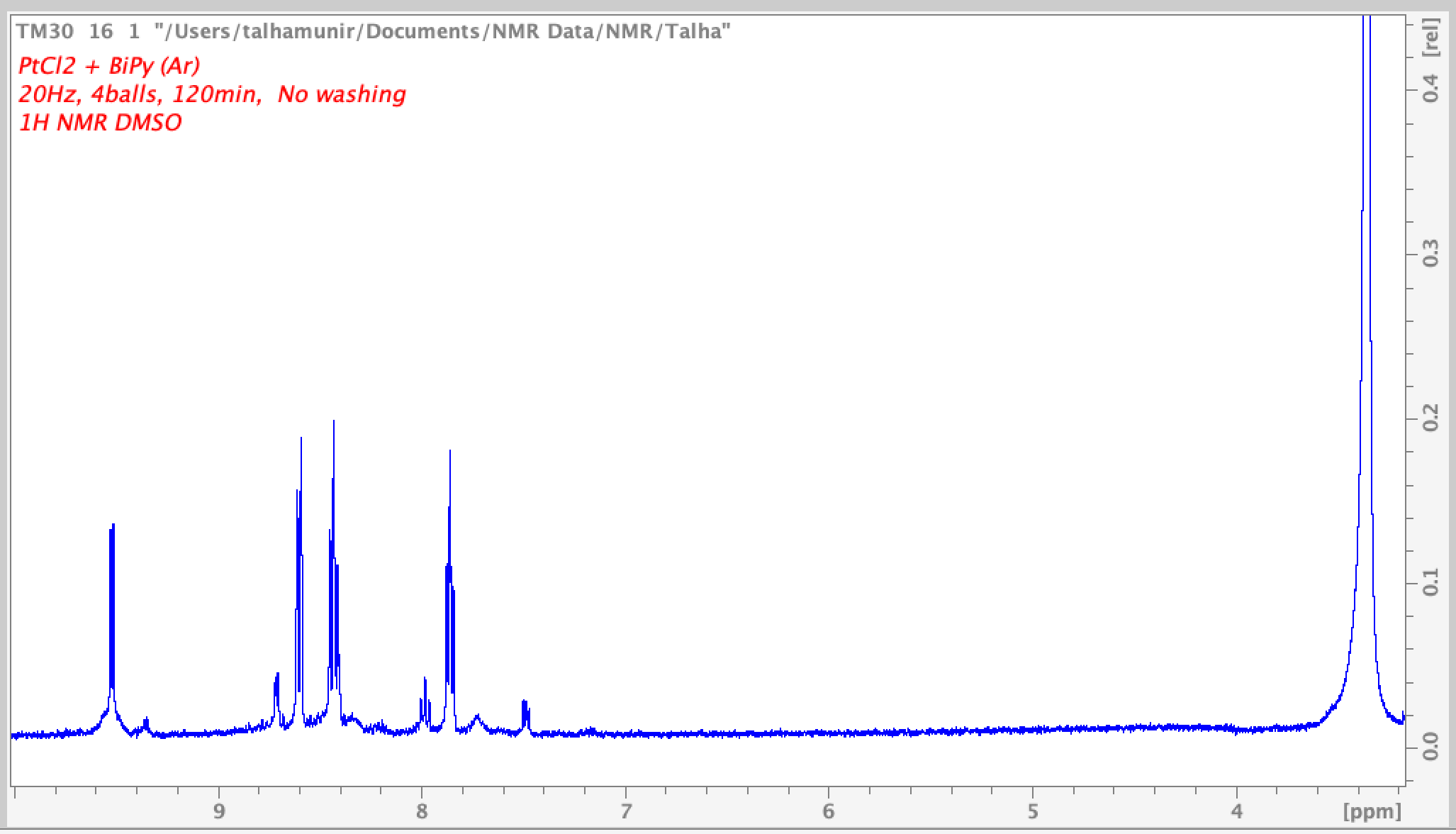


Figure S16: ^1^H-NMR Spectra of Pt(BiPy)Cl_2_ **(8)**


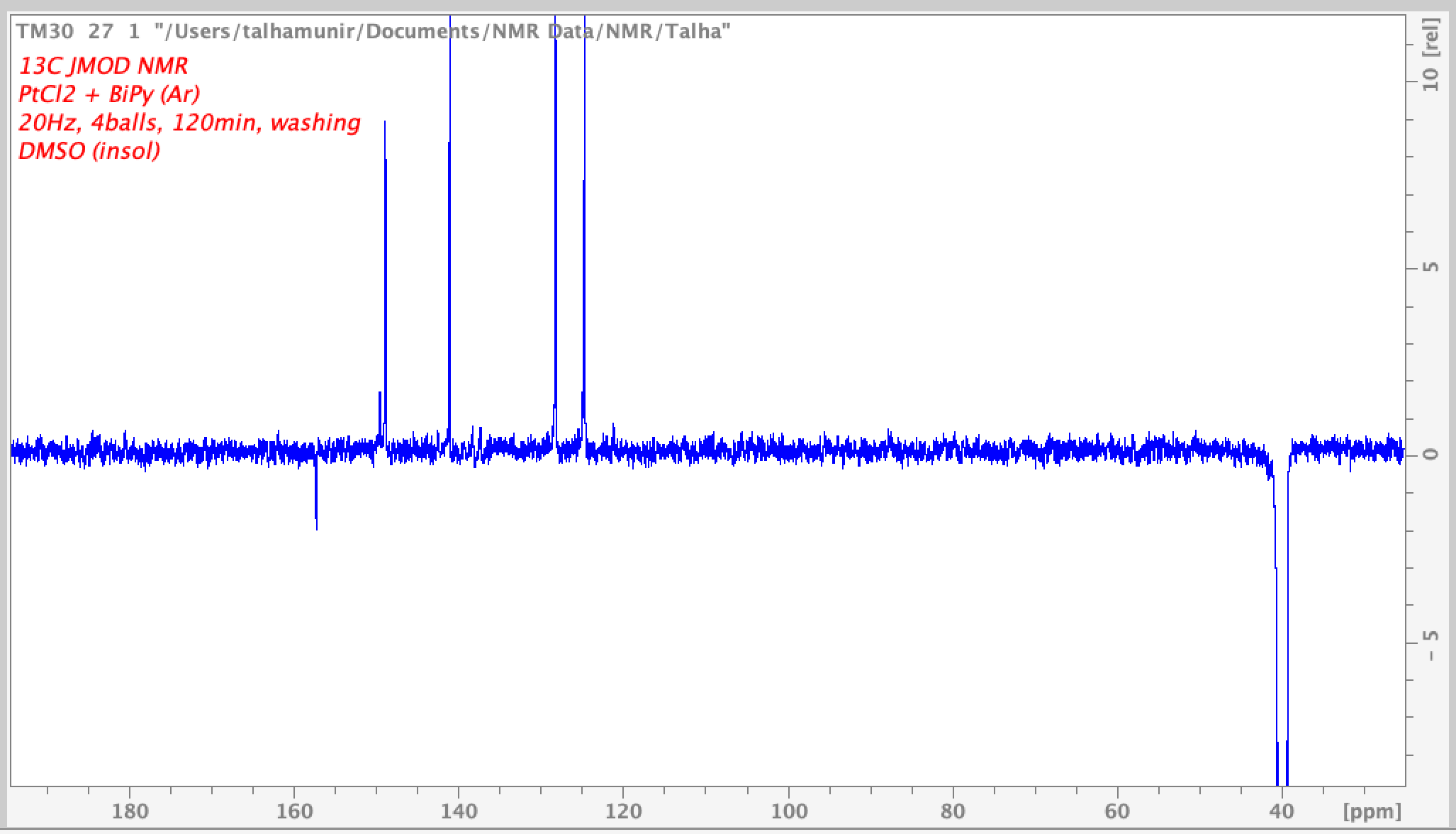


Figure S17: ^13^C-NMR Spectra of Pt(BiPy)Cl_2_ **(8)**


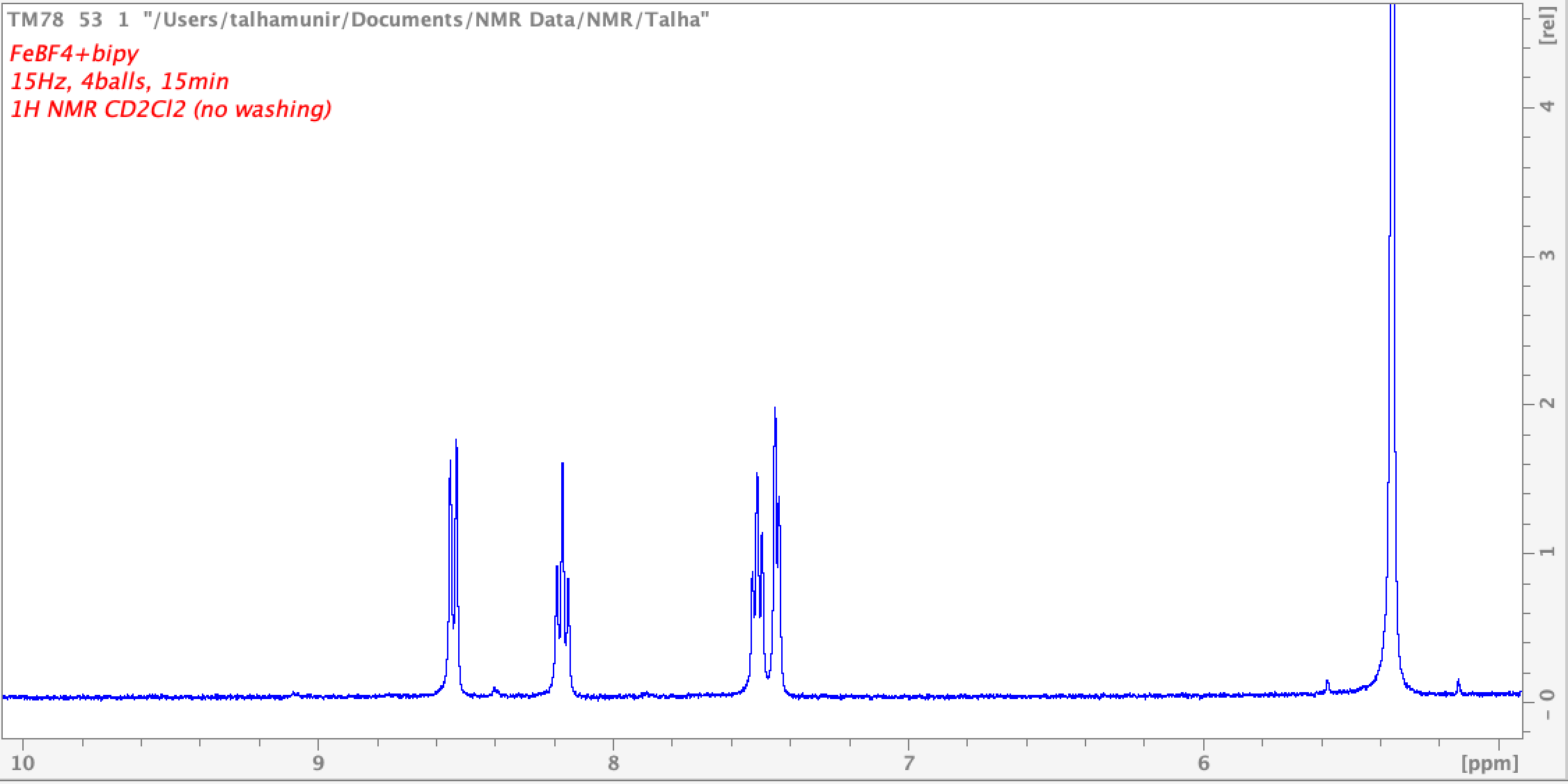


Figure S18: ^1^H-NMR Spectra of Fe(BiPy)_3_(BF_4_)_2_ **(9)**


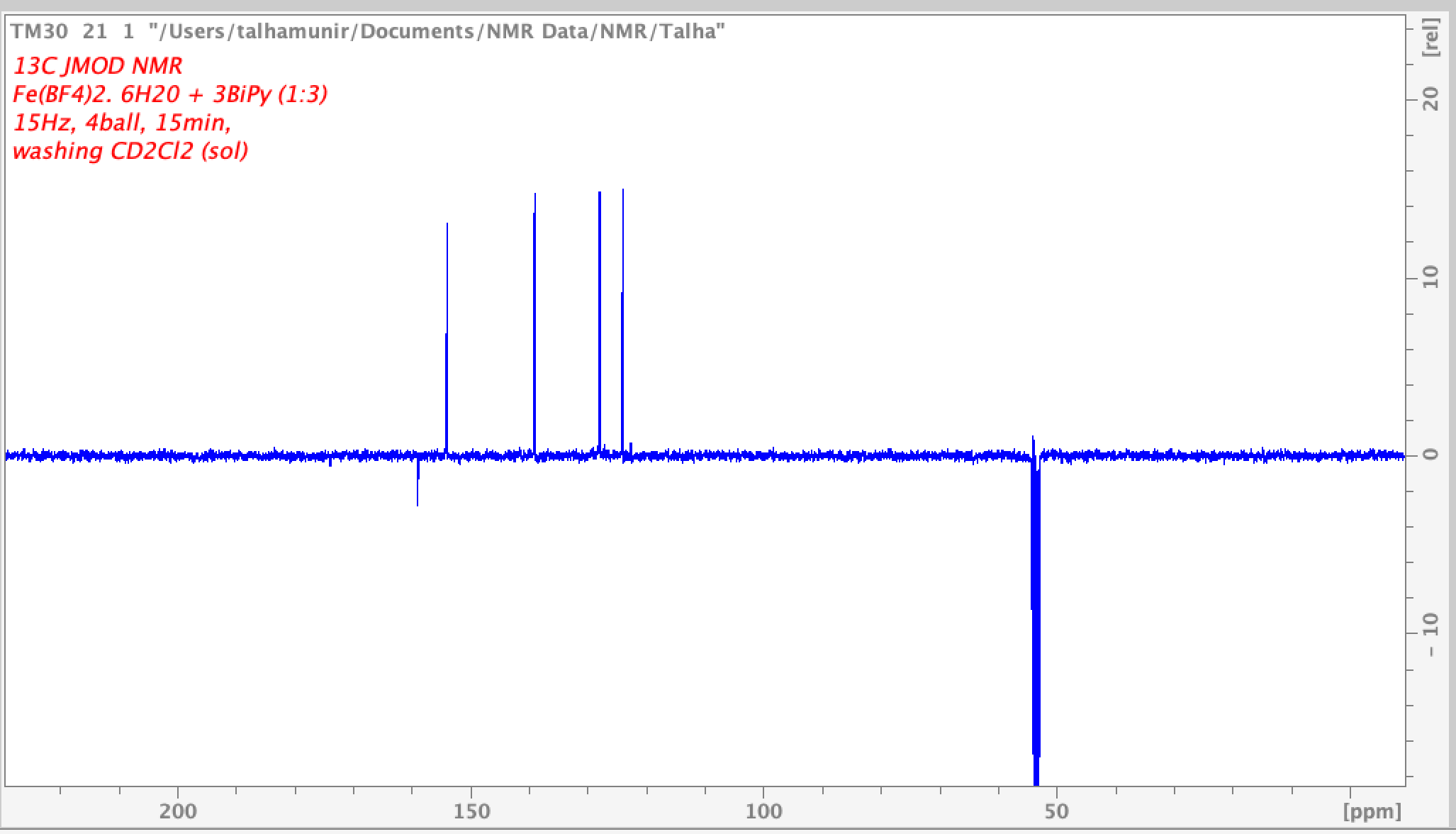


Figure S19: 13C-NMR Spectra of Fe(BiPy)_3_(BF_4_)_2_ **(9)**


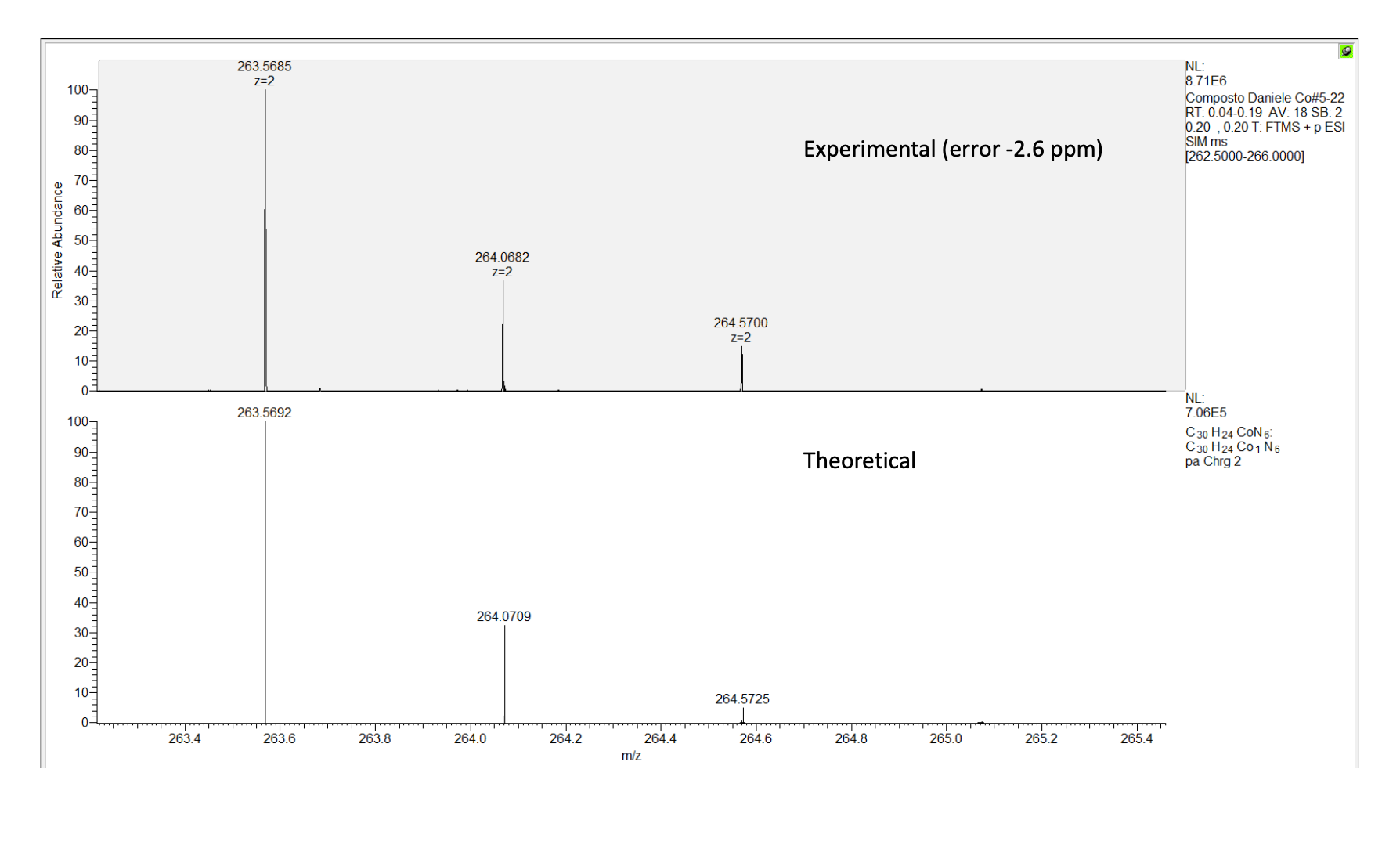


Figure S20: Mass Spectra of Co(BiPy)_3_Cl_2_ **(10)**


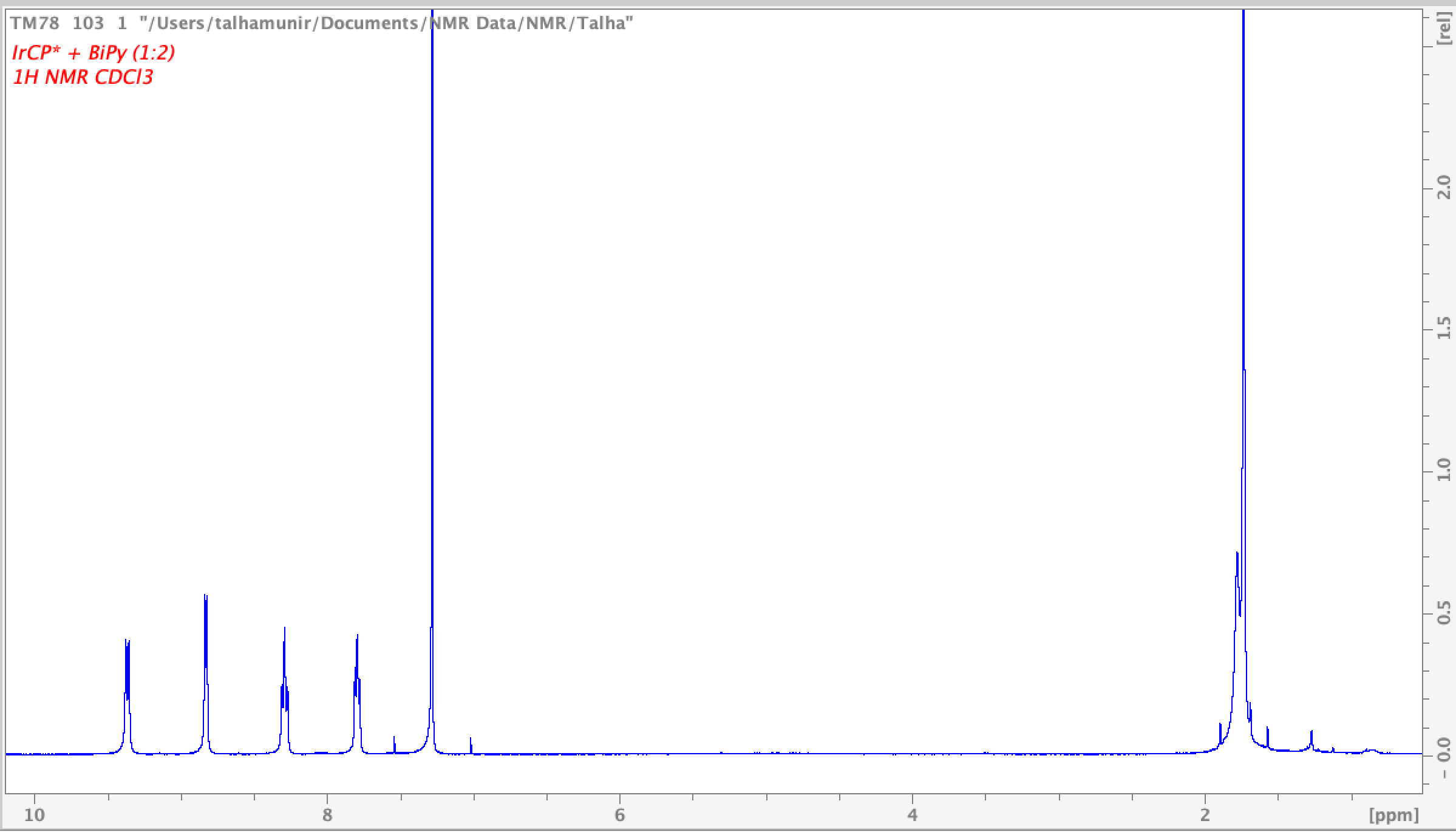


Figure S21: ^1^H-NMR Spectra of [Ir(Cp*)(BiPy)Cl]Cl **(11)**


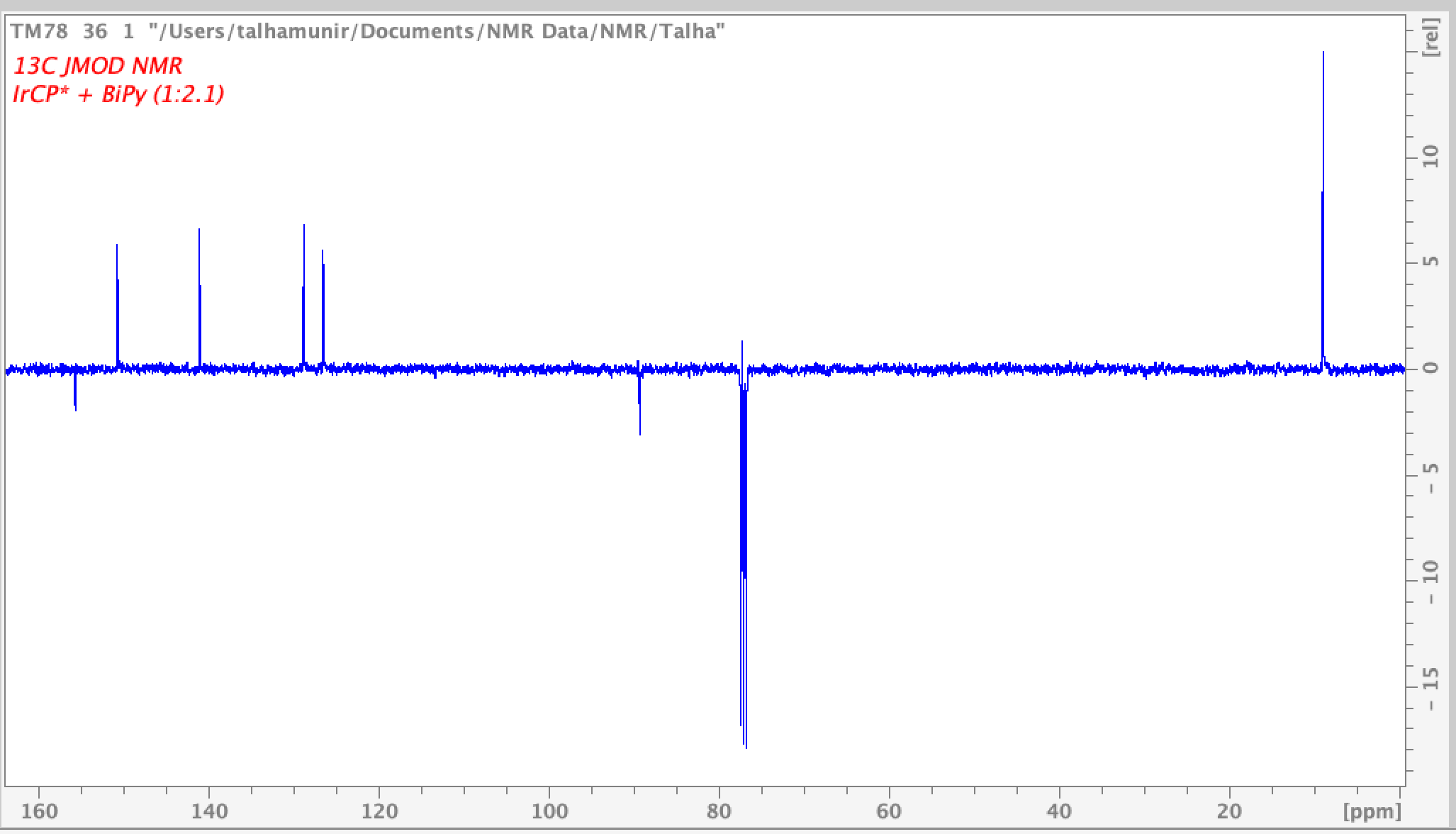


Figure S22: ^13^C-NMR Spectra of [Ir(Cp*)(BiPy)Cl]Cl **(11)**

**Part 5: XRD analysis**

XRD diffractograms were recorded on a Philips X’Pert diffractometer (equipped with a real time multiple strip detector) operated at 40kV and 40mA using Ni-filtered Cu-Kα radiation. Spectra were collected using a step size of 0.02° and a counting time of 10 s per angular abscissa in the range 5°-40°

Powder XRD patterns were recorded for (i) the complex **1** isolated from the ball mill and (ii) the same complex that had been crystallized from solution (Figure S23). The two patterns are identical, confirming that the product obtained via mechanochemistry is phase-pure and structurally equivalent to that prepared through the conventional solution-based method.

**

Figure S23: XRD profiles of compound **1** prepared via mechanochemical procedure (blue line) and conventional solution method (red line).
